# Novel Activities of Select NSAID R-Enantiomers against Rac1 and Cdc42 GTPases

**DOI:** 10.1371/journal.pone.0142182

**Published:** 2015-11-11

**Authors:** Tudor I. Oprea, Larry A. Sklar, Jacob O. Agola, Yuna Guo, Melina Silberberg, Joshua Roxby, Anna Vestling, Elsa Romero, Zurab Surviladze, Cristina Murray-Krezan, Anna Waller, Oleg Ursu, Laurie G. Hudson, Angela Wandinger-Ness

**Affiliations:** 1 Comprehensive Cancer Center, University of New Mexico Health Sciences Center, Albuquerque, New Mexico, United States of America; 2 Translational Informatics Division, Department of Internal Medicine, University of New Mexico Health Sciences Center, Albuquerque, New Mexico, United States of America; 3 Department of Pathology, University of New Mexico Health Sciences Center, Albuquerque, New Mexico, United States of America; 4 University of New Mexico Center for Molecular Discovery, University of New Mexico Health Sciences Center, Albuquerque, New Mexico, United States of America; 5 Division of Epidemiology, Biostatistics, and Preventive Medicine, Department of Internal Medicine, University of New Mexico Health Sciences Center, Albuquerque, New Mexico, United States of America; 6 Department of Pharmaceutical Sciences, College of Pharmacy, University of New Mexico Health Sciences Center, Albuquerque, New Mexico, United States of America; Beatson Institute for Cancer Research Glasgow, UNITED KINGDOM

## Abstract

Rho family GTPases (including Rac, Rho and Cdc42) collectively control cell proliferation, adhesion and migration and are of interest as functional therapeutic targets in numerous epithelial cancers. Based on high throughput screening of the Prestwick Chemical Library^®^ and cheminformatics we identified the R-enantiomers of two approved drugs (naproxen and ketorolac) as inhibitors of Rac1 and Cdc42. The corresponding S-enantiomers are considered the active component in racemic drug formulations, acting as non-steroidal anti-inflammatory drugs (NSAIDs) with selective activity against cyclooxygenases. Here, we show that the S-enantiomers of naproxen and ketorolac are inactive against the GTPases. Additionally, more than twenty other NSAIDs lacked inhibitory action against the GTPases, establishing the selectivity of the two identified NSAIDs. R-naproxen was first identified as a lead compound and tested in parallel with its S-enantiomer and the non-chiral 6-methoxy-naphthalene acetic acid (active metabolite of nabumetone, another NSAID) as a structural series. Cheminformatics-based substructure analyses—using the rotationally constrained carboxylate in R-naproxen—led to identification of racemic [R/S] ketorolac as a suitable FDA-approved candidate. Cell based measurement of GTPase activity (in animal and human cell lines) demonstrated that the R-enantiomers specifically inhibit epidermal growth factor stimulated Rac1 and Cdc42 activation. The GTPase inhibitory effects of the R-enantiomers in cells largely mimic those of established Rac1 (NSC23766) and Cdc42 (CID2950007/ML141) specific inhibitors. Docking predicts that rotational constraints position the carboxylate moieties of the R-enantiomers to preferentially coordinate the magnesium ion, thereby destabilizing nucleotide binding to Rac1 and Cdc42. The S-enantiomers can be docked but are less favorably positioned in proximity to the magnesium. R-naproxen and R-ketorolac have potential for rapid translation and efficacy in the treatment of several epithelial cancer types on account of established human toxicity profiles and novel activities against Rho-family GTPases.

## Introduction

The Ras-homologous (Rho) family of small GTPases (Rac, Rho and Cdc42) are key regulators of actin reorganization, cell motility, cell-cell and cell-extracellular matrix adhesion as well as of cell cycle progression, gene expression and apoptosis [1–7]. These critical functions place Rho family GTPases in the midst of normal and pathophysiological processes across tissue and organ systems [8–10]. In addition, the activities regulated by Rho-family GTPases are intimately linked to the development and progression of cancer [11–14].

In many human cancers, one or more Rho-family members are over-expressed or mutant and hyperactivity is often associated with increased aggressiveness and poor patient prognosis [10, 15–20]. Stimulation of downstream targets and signaling pathways are linked to tumor growth and survival, invasion and metastasis [5, 15, 21, 22]. The specific mechanisms by which Rho-family GTPases influence transformation and tumor progression are still emerging [1, 3, 10, 23], yet the clinical and experimental evidence place Rac1 and Cdc42 within the metastatic cascade and provide an imperative for focused attention on these proteins as potential therapeutic targets in cancer that has not yet been realized.

Rho-family GTPase activities are tightly regulated by the GDP/GTP binding cycle and localization between cytoplasm and membrane compartments [24]. GTPase signaling may be inhibited by many mechanisms including disruption of the C-terminal isoprenylation which is required for correct intracellular localization and function [25], competitive inhibition by guanine-mimetic analogues that interfere with the active GTP bound state [26], disruption of the activity of Rho-specific activator proteins (i.e. GEFs) or perturbation of effector coupling thereby blocking downstream signaling [5, 9, 24]. Despite the promise of such small molecules in cell-based assays [27–30], few have been studied in a preclinical context [31–34] and none have been translated into a clinical context.

Our studies were motivated by the more rapid clinical translation afforded by repurposing/repositioning known drugs for new targets [35]. To this end we conducted high throughput screens of the Prestwick Chemical Library^®^ of off patent and FDA approved drugs and drug-like small molecules for inhibitors and activators of small GTPases. A similar approach identified Ras signaling inhibitors [36, 37]. Through a combination of *in vitro* and *in silico* screening we identified the R-enantiomers of select nonsteroidal anti-inflammatory drugs (NSAID) naproxen and ketorolac as Rac1 and Cdc42 inhibitors whereas many other related NSAIDs were inactive. The S-enantiomers of naproxen or ketorolac, well known as highly active cyclooxygenase inhibitors, displayed little or no activity against the GTPase targets thereby illustrating stereoselectivity. Although it has been long recognized that R-enantiomers of NSAIDs are poor inhibitors of cyclooxygenase activity [38–46], relatively little is known about potential pharmacologic activities or targets for these R-enantiomers [47–49]. Our findings suggest that specific NSAID R-enantiomers have novel activity as modulators of Rac1 and Cdc42.

## Materials and Methods

### Materials

GST-tagged GTPases were either obtained from Cytoskeleton or purified as previously described [50]. Cyto-Plex^™^ microspheres (4.0 μm) used for screening assays were from Thermo Fisher Scientific; larger, preactivated 13 μm Superdex beads used for more detailed analytical studies were custom synthesized by Amersham Biosciences and conjugated with GSH as previously described [26]. BODIPY^®^ FL GTP and BODIPY^®^ FL GDP were from Life Technologies. The automated HyperCyt^®^ delivery system invented at UNM was used with the CyAn^™^ADP flow cytometer from Beckman Coulter. The Prestwick Chemical Library^®^ (2007) of off patent drugs and drug-like compounds contained 880 small molecules (Prestwick Chemical, Washington DC, USA). Compounds for secondary dose response assays were obtained as follows: Acetylsalicylic acid (A5376, Sigma-Aldrich, St. Louis, MO, USA), Celecoxib (Prestwick Chemical Library^®^, Washington DC, USA), (S)-(+)-Ibuprofen (I106, Sigma-Aldrich), Ketoprofen (K2012, Sigma-Aldrich), Fenoprofen (F1517, Sigma-Aldrich), R-naproxen (Prestwick Chemical Library^®^), S-naproxen (N5160,Sigma-Aldrich), 6-methoxy-2-napthalene acetic acid (6-MNA) (70620, Cayman Chemical, Ann Arbor, MI, USA), Sulindac (Prestwick Chemical Library^®^), Sulindac sulfide (Prestwick Chemical Library^®^), Valdecoxib (Prestwick Chemical Library^®^). Compounds for cell-based studies were obtained from the following sources: R-naproxen (Acme Bioscience #A5026, Palo Alto, CA), S-naproxen (Sigma #N5160, St Louis, MO), 6-Methoxynaphthalenic Acid (Cayman Chemical #70620, Ann Arbor, MI), NSC23766 (Santa Cruz Bio #sc-204823, Santa Cruz, CA), R-ketorolac (K235600, Toronto Research Chemicals), S-ketorolac (K235605, Toronto Research Chemicals), racemic ketorolac (K1136, Sigma-Aldrich). Human epidermal growth factor (EGF) was from BD Biosciences (San Jose, CA). Specific reagents used for individual applications are specified below.

### Cell Lines

Mouse NIH/Swiss 3T3 fibroblasts (CRL-1658) were from ATCC (http://www.atcc.org) and maintained in DMEM media (Invitrogen, Carlsbad, CA, USA). HeLa T4+ were from NIH AIDS Reagent Program (catalog number 154) and maintained in DMEM, 10% FCS and 500 μg/ml G418 [51]. Immortalized human ovarian cancer (OvCa429 and OvCa433) cell lines were a kind gift of Dr. Robert C. Bast, Jr. (University of Texas MD Anderson Cancer Center) [52], and were maintained in MEM supplemented with 10% fetal bovine serum, 50 U/mL and 50 μg/mL of penicillin/streptomycin, respectively, 1 mM sodium pyruvate and 2 mM L-glutamine (Sigma-Aldrich, St. Louis, MO) in a CO_2_ incubator at 37°C.

### Ethics Statement

Immortalized human ovarian cancer cell lines (OvCa429 and 433) and HeLa T4+ are previously published [51, 52].

### Multiplex Screening Procedures and Dose Response Assays

For multiplex analyses of small GTPases, 4 μm diameter glutathione-beads (GSH-beads) distinguished by seven different intensities of red fluorescence were used according to our previously described protocols [30, 53, 54]. The GST-GTPases included the following eight Ras-related small GTPases: Rab2 wt, Rab7 wt, Cdc42 wt, Cdc42Q61L activating mutant, H-Ras wt, H-RasG12V, Rac1 wt and Rac1Q61L activating mutant. Briefly, Prestwick Chemical Library^®^ compounds were tested at 10 μM compound and 1% DMSO final concentrations. After a 10 min pre-incubation with the compound BODIPY- FL-GTP (100 nM final) was added to each well and bead associated fluorescence was measured. Sample analysis was conducted with a HyperCyt^®^ high throughput flow cytometry platform. Flow cytometric light scatter at 488 nm and fluorescence emission at 530 +/− 20 nm (FL1) and 665 +/− 10 nm (FL8) were collected on a Cyan ADP flow cytometer (Beckman Coulter, Fullerton, CA). IDLQuery software was used to determine the compound activity in each well. Gating based on forward scatter (FS) and side scatter (SS) parameters was used to identify singlet bead populations. Gating based on FL8 emission distinguishes the beads coated with different proteins, and the median FL1 fluorescence per bead population were calculated. A compound was considered a “potential active” if the change in BODIPY-FL-GTP binding differed greater than 20% from that measured in DMSO treated controls.

Test compounds identified for follow-up after the primary screen were tested at dilutions ranging from 10 nM to 100 μM final concentration against individual GTPases in multiplex and single-plex assays. Compounds tested in dose response assays were serially diluted 1:3 a total of eight times from a starting concentration of 10 mM giving a 9-point dilution series in DMSO.

### G-trap Flow Cytometric Effector Binding Assay to Measure GTPase Inhibitory Activity of Compounds in Cells

HeLa cells were serum-starved for 2 h in growth medium lacking fetal bovine serum followed by a 2 h treatment with increasing concentrations (30–1000 μM) of 6MNA, R- or S-naproxen (including 30 μM, 100 μM, 300 μM, 1000 μM), and finally stimulation with 100 ng/ml EGF for 2 min. HeLa cells were serum-starved for 2 h in growth medium lacking fetal bovine serum followed by pretreatment with 10 μM R-, S-, racemic ketorolac or 0.1% DMSO for 1 h and stimulation with epidermal growth factor for 2 min. Cdc42 specific inhibitor CID2950007/ML141 (10 μM) and Rac1 specific inhibitor NSC23766 (10 μM) served as positive controls. Treated and vehicle-treated cells were lysed with RIPA buffer (50 mM Tris-HCl, pH 7.4, 150 mM NaCl, 0.25% (w/v) deoxycholate, 1 mM EDTA, 1 mM NaF, 1 mM PMSF, 1 mM Na_3_VO_4_, 1% (v/v) NP40, and protease inhibitor cocktail consisting of chymostatin, leupeptin, antipain and pepstatin) and insoluble debris removed by centrifugation. Supernatants were incubated for 1 h with GST-effector domains (PAK1- PBD for Cdc42 and Rac1) immobilized on GSH beads. Antibodies specifically directed against Cdc42 and Rac1 were used to quantify the amount of active (GTP-bound) GTPase on the beads. Both primary and secondary antibodies were incubated with the beads for 1 h. Fluorescence intensity (mean channel fluorescence, MCF) was measured by flow cytometry (Accuri C6). GTPase activity was calculated by (MCF of sample group—MCF of unstimulated group) / MCF of stimulated group.

GST fusion PAK-1 PBD (14–864) was from Millipore. The primary antibodies were anti-Cdc42 (Santa Cruz sc-8401) and anti-Rac1 (BD Transduction Labs 610650). The secondary antibody was Alexa Fluor 488 donkey anti-mouse lgG (Life Technologies A21202). Testing antibody reactivities against effector beads without added cell lysates or effector beads expected to bind different GTPases served as controls to validate antibody specificities [55].

### Cyclooxygenase Assays

The effects of compounds on the activity of the human COX-1 and COX-2 were quantified by measuring the formation of prostaglandin E2 (PGE2) from arachidonic acid using recombinant enzyme isolated from transfected Sf-9 cells. The analyses were conducted by CEREP Laboratories USA (Redmond, WA and Le bois l'Evêque, France) using the method of Glaser et al [56]. Briefly, The test compound, reference compound or water (control) were pre-incubated for 20 min at room temperature with the enzyme (≈ 5 μg) in a buffer containing 90 mM Tris-HCl (pH 8.0), 1.98 mM phenol and 1.02 μM hematine. The reaction was initiated by adding 4 μM arachidonic acid and the mixture incubated for 5 min at room temperature. For basal control measurements, arachidonic acid was omitted from the reaction mixture. The reaction was stopped by the addition of 1 M HCl then 1 M Tris/HCl (pH 8.0) followed by cooling to 4°C. The fluorescence acceptor (d2 labeled PGE_ß_) and the fluorescence donor (anti-PGE_2_ antibody labeled with europium Cryptate) were then added. After 120 min, the fluorescence transfer corresponding to the amount of residual PGE_2_ was measured at λ_ex_ 337 nm, λ_em_ 620 nm and λ_em_ 665 nm using a microplate reader (Envision, Perkin Elmer). The enzyme activity was determined by dividing the signal measured at 665 nm by that measured at 620 nm (ratio). The results are expressed as a percent inhibition of the control enzyme activity and compared against published literature (see Table C in S1 File). The standard inhibitory reference compounds are diclofenac and NS398 for COX-1 and COX-2, respectively.

### GLISA

Swiss 3T3 cells were continuously passaged at subconfluence and used to monitor the capacity of compounds to block stimulus-mediated activation of Cdc42 or Rac1 *in vivo*. Commercial GLISA kits customized to capture activated Cdc42, Rac1, or RhoA from cell lysates were used per manufacturer’s instructions (Cytoskeleton, Inc., Denver, CO, USA.). Cells were serum-starved by sequentially removing serum over a 3-day period and treated with either 0.1% DMSO or test compound at the concentrations and duration as indicated in the legends. Subsequently, samples were treated with 10 ng/ml (OvCa433) or 100 ng/ml EGF (3T3 and HeLa cells) for 2 min to activate Cdc42 or Rac1. DMSO-treated cells were left either unstimulated to determine base-line GTPase activation or stimulated without drug treatment to determine maximal GTPase activation. The 2 min time point was determined from a time course of EGF-dependent Rac1 activation to establish the time reflecting maximal GTPase activation in Swiss 3T3 and OvCa433 cell lines.

### Rac1 and TIAM1 Immunolocalization

OvCa433 were plated on coverslips, treated with 300 μM NSAIDs for 1 h, fixed with 3% paraformaldehyde and immunostained for Rac1 or Tiam1. Actin was visualized with rhodamine phalloidin from Invitrogen (F432). Cells were imaged on a Zeiss LSM510 using a 63x objective and quantified using Image J.

Primary and secondary antibodies were as follows: anti-Rac1 (mouse mAb IgG2b raised against human Rac1, validated for Western blot and immunofluorescence analyses to detect human, mouse, rat, dog and chicken proteins, BD Transduction Labs 610650), Tiam1 (affinity purified rabbit pAb raised against a peptide in the C-terminus of murine Tiam, validated for Western blot and immunofluorescence analyses to detect mouse, rat and human proteins, sc-872, Santa Cruz Biotechnology, Inc.), anti-rabbit (711-175-152) and anti-mouse (715-175-150) secondary antibodies conjugated to Cy5 were from Jackson Immunoresearch. Rac1 and Tiam1 antibodies were confirmed in our hands to recognize single protein species of the predicted molecular weight by immunoblot.

### SDS-PAGE and Immunoblot Analyses

OvCa433 cells were seeded in 60 mm dishes at ~40,000 cells/mL in complete medium. When cells reached 80% confluence the media was changed to 0.1% BSA/MEM with or without 300 μM S- or R-naproxen for 24 or 48 h. Cells were stimulated with EGF at a final concentration of 1 nM for 10 min. Cells were lysed in a RIPA buffer consisting of 50 mM Tris pH 7.4, 1% NP-40, 0.25% sodium deoxycholate, 150 mM NaCl, 1 mM EDTA, 1 mM PMSF, 1 mM sodium vanadate, 10 mM sodium fluoride, 1 μg/mL each of pepstatin, aprotinin and leupeptin, 10 mM sodium pyrophosphate and 10 mM beta-glycerophosphate. The lysates were sonicated and protein content measured using a BCA protein assay (Thermo Fisher Scientific, Inc.). The proteins were resolved by SDS PAGE and then transferred to PVDF membranes and blocked in 3% BSA/TBS-T (50 mM Tris pH 7.6, 150 mM NaCl, 0.05% Tween 20) solution for 1 h or 1% BSA/TBS-T solution overnight prior to probing with the following primary antibodies.

Anti-phosphorylated-EGFR (rabbit mAb IgG, raised against a synthetic phospho-peptide corresponding to residues surrounding Tyr1068 of the human EGF receptor, validated for Western blot and immunofluorescence analyses to detect human, mouse, monkey, and rabbit proteins, #3777, Cell Signaling Technology), anti-phosphorylated-MAPK (Erk1/2) (mouse mAb IgG1, raised against a synthetic phospho-peptide corresponding to residues surrounding Thr202/Tyr204 of the human p44 MAP kinase, validated for Western blot and immunofluorescence analyses to detect human and mouse, among other proteins, #9106, Cell Signaling Technology), anti-pan-ERK (mouse mAb IgG2a, validated for Western blot and immunofluorescence analyses to detect human and mouse among other proteins, 610123, BD Transduction Laboratories), anti-EGFR antibodies (mouse mAb, validated for Western blot to detect human protein, Santa Cruz biotechnology, Santa Cruz, CA), anti-Rac1 and anti-Tiam1 were as described for immunolocalization studies.

### Boyden Chamber Cell Migration Assay

Cell migration assays were performed on modified Boyden chambers as described [57]. OvCa cells (5 x 10^4^) were suspended in serum-free media (0.1% BSA in MEME) with or without varying concentrations (0–300 μM) of R-naproxen, S-naproxen, 6-MNA, and NSC 23766. Cells were allowed to migrate for 48h through cell culture inserts with 8 μm pores (Becton Dickinson Labware) for 48h at 37°C. Non-migratory cells were removed. Migratory cells were stained with 0.2% crystal violet in 10% ethanol and membranes with cells were stained with DAPI (Vector Laboratories #H-1200 Vectashield with DAPI, Burlingame, CA). To quantify migration, three independent fields of migratory cells per well were photographed under phase-contrast and fluorescence microscopy. The number of cells per field was counted and an average of the three determinations was obtained for each chamber. Each migration assay was performed a minimum of three times.

### Invadopodia Assays and Microscopic Imaging

Invadopodia assays were performed as described previously [58–60] and modified to incorporate drug treatment and EGF stimulation as follows. Briefly, sterile microscopy grade coverslips (18Cir-1; Fisher) were coated with 0.01% collagen at 6–10 μg/cm^2^ in PBS pH 7.2 for 3 h at 37°C and allowed to dry overnight. Collagen (calf skin C8919; Sigma-Aldrich) was fixed for 4 h at 37°C with 4% gluteraldehyde in PBS. Fibronectin (human plasma F2006; Sigma-Aldrich) was dissolved at 1 mg/ml in water and then diluted in PBS and used at 1–5 μg/cm^2^ for coating coverslips. FITC-labeled fibronectin was prepared according to manufacturer’s instructions (FluoReporter^®^ FITC protein labeling kit 6434, Invitrogen Life Technologies) and excess unconjugated fluorophores were removed by gel filtration. Final FITC-fibronectin concentration of 1 mg/ml and total of 20 μl/coverslip was placed on parafilm and collagen coated coverslip placed carefully face down of the drop taking care to avoid trapping air bubbles and allowed to set for 3 h in a humidified chamber. Coated coverslips were washed with 70% ethanol to sterilize. Coverslips with a flawless even FITC-fibronectin coating were transferred to 6-well tissue culture wells and seeded with OvCa429 cells plated at 2.5x10^4^ cells/well in serum free medium. After 12 h in culture, cells were treated +/- 10 ng/ml EGF +/- individual drugs (6-MNA, R- or S-naproxen) at 300 μM final concentration which is equivalent to human serum plasma levels at clinically relevant dosages [61, 62]; 1:1000 dilution from sterile stock in water. At varying time points (0–24 h) samples were fixed with 3% paraformaldehyde in PBS, permeabilized with 0.1% Triton X-100 and immunostained for Rac, Cdc42, among other markers. Actin was visualized with rhodamine phalloidin (diluted 1:50 in 1% BSA/PBS^+^). Cells were imaged on a Zeiss LSM 510 with 20x, and 63x oil immersion objectives. Cell morphologies were characterized based upon extent and symmetry of filopodia and lamellipodia and categorized as nonadherent, extended or resting phenotype. Invadopodia were identified and quantified based on presence of Cdc42, actin and cortactin and loss of FITC staining.

Primary and secondary antibodies were as follows: anti-Rac1 (as detailed under Rac and Tiam Immunolocalization above, BD Transduction Labs 610650), anti-Cdc42 (rabbit pAb raised against synthetic peptide corresponding to human Cdc42 amino acid residues 141–154 from Stressgen KAS-GP007), secondary antibodies and rhodamine phalloidin as described under Rac1 and Tiam1 Immunolocalization.

### Ligand-Based Virtual Screening

Similarity coefficients were computed using the Tanimoto coefficient [63], using R-Naproxen (first confirmed hit) as a query. 3D (three-dimensional) similarity was computed using ROCS [64–67] starting from 100 distinct, low-energy conformers enumerated with OMEGA [68–71]. 2D similarity was computed using our in-house implementation of MDL keys [72]. Ligand-based virtual screening (LBVS) focused on 39 NSAIDs; to supplement the 20 Prestwick Chemical Library^®^ drugs, an additional 19 were added to match the list of marketed NSAIDs studied for cyclooxygenase 1 and 2 inhibition, respectively [73, 74]. Observing a protocol that had led to the identification of the first reported G-protein estrogen receptor agonist [75], the LBVS composite score was weighted as follows: 40%, 2D-based Tanimoto coefficient; 50%, 3D-based (shape) similarity; and 10%, scaled Color Score. The Color Score estimates pharmacophore similarity using the implicit Mills-Dean approximation [76].

### The Alpha-Methyl Carboxylate Hypothesis

LBVS ranked 6-MNA, the active metabolite of the non-racemic NSAID nabumetone, as well as nabumetone itself as most similar to R-Naproxen. On this basis, 6-MNA was selected and tested, but lacked activity against Rac1 and Cdc42. As illustrated in our decision workflow, this was the basis of the alpha-methyl carboxylate (α-Me-COOH) hypothesis, which led to the identification of racemic ketorolac as the primary candidate NSAID for further studies, since it is approved for human use. An additional set of alpha-methyl carboxylates and di-methyl-carboxylates that are approved drugs were subjected to an LBVS counter-screen. In this case, searches were conducted for drugs with high scores compared to R-ketorolac or R-naproxen, as well as low scores when compared to S-ketorolac or S-naproxen.

### Docking of NSAIDs to the Nucleotide Binding Site of Rac1 and Cdc42

Docking pose predictions are based on the mechanism of GEF mediated nucleotide displacement garnered from structural studies of Cdc42 and DOCK9 ^DHR2^ GEF [77], as well as from the Rac1 and DOCK2^DHR2^ GEF complex [78]. We also considered novel allosteric pockets on Rho GTPases that were identified with molecular dynamics simulations [79] and determined that additional experimental validation is required. PDB ID 2WMN (human CDC42 in complex with human Dedicator of Cytokinesis protein 9, DOCK9, GEF) was used for making docking predictions for NSAID-Cdc42 complexes. According to Yang [77], Val1951 in DOCK9 alpha10 helix mediates Mg^2+^ exclusion and reduces nucleotide binding affinity to the GTPase pocket. NSAIDs were docked onto the nucleotide binding pocket of Cdc42 using Val1951 in DOCK9 to select the initial docking pose. PDB ID 2YIN (human Rac1 in complex with human DOCK2 GEF) was used for making docking predictions for NSAID-Rac1 complexes. All docking studies were performed with Molecular Operating Environment (MOE) software package (Chemical Computing Group, Montreal, Quebec Canada). Docking poses were scored based on the ability of carboxyl moieties on the NSAIDs to interact with and neutralize the Mg^2+^ positive charge leading to reduced nucleotide affinity or Mg^2+^ displacement.

### Statistical Analyses

Z’ scores were calculated and served as a statistical measure of screening and dose response data. Excel and GraphPad Prism were used for statistical analyses, binding and sigmoidal dose response data fitting. One-way ANOVA with Dunnett’s or Tukey’s multiple comparison post-test were used with specific details of method and results given in each individual figure legends.

## Results

### High-throughput screening of off patent drugs against Rho-family GTPases

**Fig 1:**
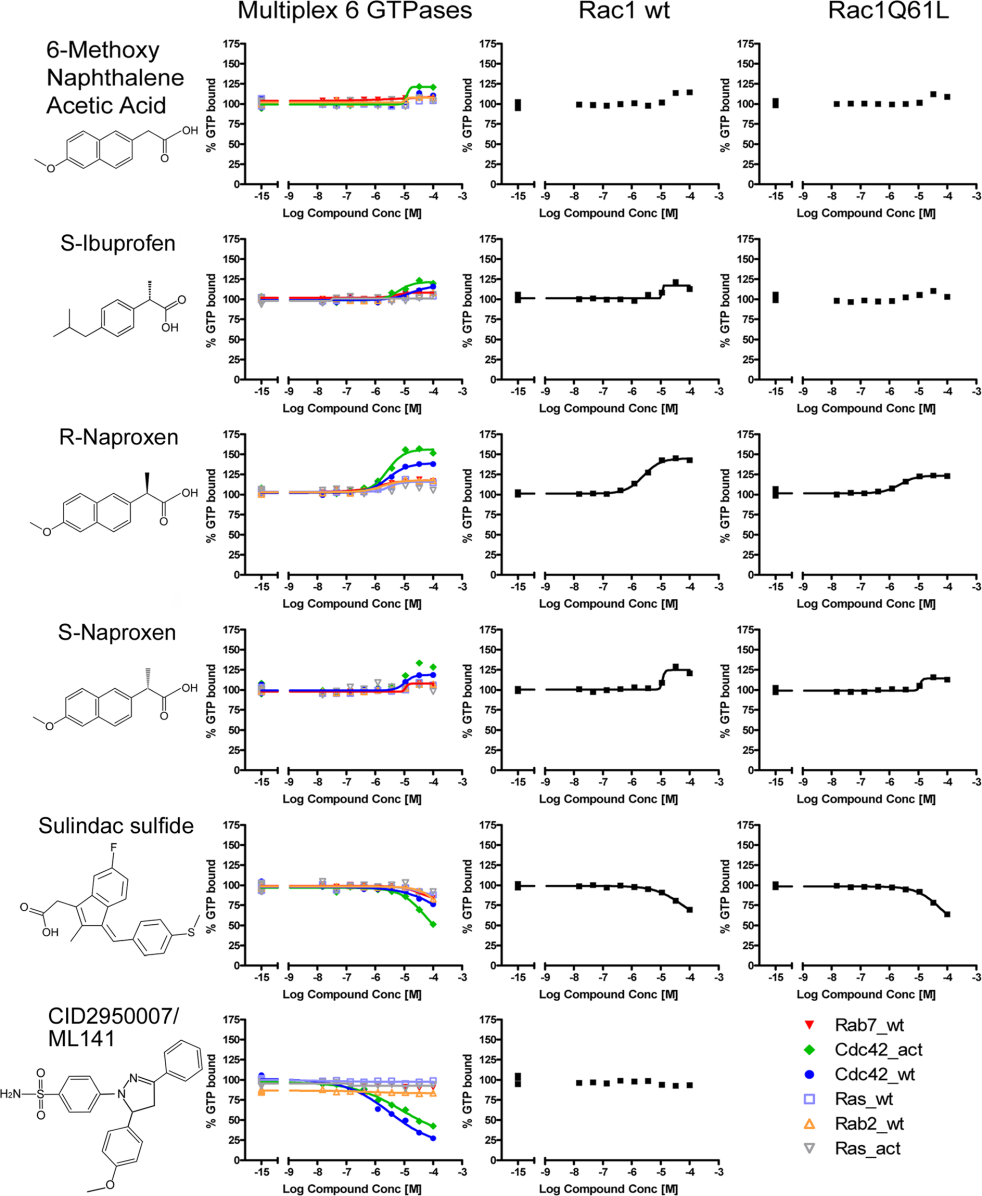
Effects of Prestwick Chemical Library^®^ compounds on guanine nucleotide binding by Ras-related GTPases. Tests of 888 compounds identified select NSAIDs as active against guanine nucleotide binding measured as the % of BODIPY-GTP bound in the presence of added compound relative to DMSO treated controls. Dose response assays on eight Ras-related identified R-naproxen as having selective activity against multiple Rac and Cdc42 GTPases. CID2950007/ML141 is a characterized Cdc42 selective inhibitor that served as a positive control. Multiplex dose response assays conducted on eight Ras-related GTPases, under the same conditions as primary screens with Z’ ranging from 0.62 to 0.84, identified R-naproxen as having selective activity against multiple Rac and Cdc42 GTPases.

A high-throughput, multiplex screen was conducted against small GTPase targets representing members of the Ras, Rho and Rab small GTPase subfamilies. The primary screen was performed on the 2007 version of Prestwick Chemical Library^®^, which contained 888 compounds of which 24 are classified as NSAIDs (Table A in S1 File). Z’ scores for the primary screen were very good across all targets, ranging from 0.62 to 0.83, with average standard deviation of 0.13. Actives in the primary screen along with related compounds were retested in multiplex, dose response assays as previously described [53, 54, 80, 81]. Dose response assays (10 nM to 100 μM of each compound) were conducted on a total of 11 NSAIDs (4 identified in the primary screen, with 7 additional compounds considered to be chemically similar to the GTPase active compounds identified in primary screens). Compound interaction was defined by changes in BODIPY-GTP-binding by greater than 20% on any one of the eight GTPases in multiplex (Cdc42 wild-type (wt), Cdc42Q61L (act), Rab2, Rab7, Ras wt, RasG12V (act)) or single-plex (Rac wt, Rac Q61L and GST-GFP) assays. Only four NSAIDs (R-naproxen, S-ibuprofen, ketoprofen and fenoprofen) were considered positive in the primary screen, while the remainder were inactive (Figure A in S1 File). In the confirmatory screens, R-naproxen, S-ibuprofen, S-naproxen, and sulindac sulfide showed varying activity levels, while all other tested NSAIDs were inactive at concentrations up to 100 μM (Fig 1, Table B in S1 File). These data indicate that interaction with small GTPases is not a general property of NSAIDs.

Notably, additional confirmatory dose response assays, conducted using different conditions affirmed the observed enantiomer-selective differences. R-naproxen was the sole NSAID with an EC_50_ value in the range of 3–18 μM (Table B in S1 File, Figure B in S1 File). The S-enantiomer of naproxen was 10–20 times less active than the R-enantiomer, and the closely related 6-methoxy-2-naphthalene acetic acid (6-MNA, active metabolite of the NSAID nabumetone that lacks the chiral center of naproxen) was inactive (less than 20% effect at 0.1 mM) against all GTPases and had only weak activity against the constitutively active Cdc42 mutant (Table B in S1 File, Figure B in S1 File). Note: similar to R-naproxen we have identified other compounds that were picked up as stimulators in the HTS [82] and discuss this point in greater detail in the Supplemental text. S-ibuprofen was inactive except against the constitutively active Cdc42 mutant. Interestingly, celecoxib and valdecoxib were also inactive despite some structural similarity to a novel Cdc42 specific guanine nucleotide binding inhibitor—CID2950007/ML141 –with μM inhibitory activity (Table B in S1 File, Figure A in S1 File) [30, 54, 81]. Thus, the GTPase inhibitory activities of R-naproxen and NSAID related compounds are likely due to unique structural features of these compounds.

S-naproxen is a non-selective inhibitor of cyclooxygenase (COX)-1 and -2 [39, 83–85] and displays enantiomeric selectivity for inhibition of COX-1 and COX-2 with the S-enantiomer having significantly greater potency than the R-enantiomer in *in vitro* assays [39] and in inflammatory models *in vivo* [84]. We confirmed the enantiomer selectivity for inhibition of COX-1 and COX-2 in biochemical assays; observed two orders of magnitude difference in EC_50_ values for R- and S-naproxen and >2 orders of magnitude difference for R- and S-ketorolac in agreement with published reports (Table C in S1 File). The quantitatively derived eudismic ratio (EC_50_/EC_50_) [86] of S-naproxen and S-ketorolac, relative to their R-enantiomers, is 100 for cyclooxygenases and further indicates a large pharmacologic activity difference between the enantiomers of these NSAIDs.

Structural studies conducted with S-naproxen established the importance of the (*S*)-α-methyl group of naproxen as a critical determinant of naproxen interaction with COX enzymes and it could not be eliminated or replaced by larger substituents [39]. Based on evidence for this (*S*)-α-methyl group in stereoselectivity of naproxen, and absence of GTPase binding to 6-MNA lacking the methyl group (Fig 1, Table B in S1 File), cell based assays were performed to probe potential enantiomeric differences in activity against Rac1 or Cdc42.

### Enantiomer-selective inhibition of Rac1 and Cdc42 activity in cells

**Fig 2:**
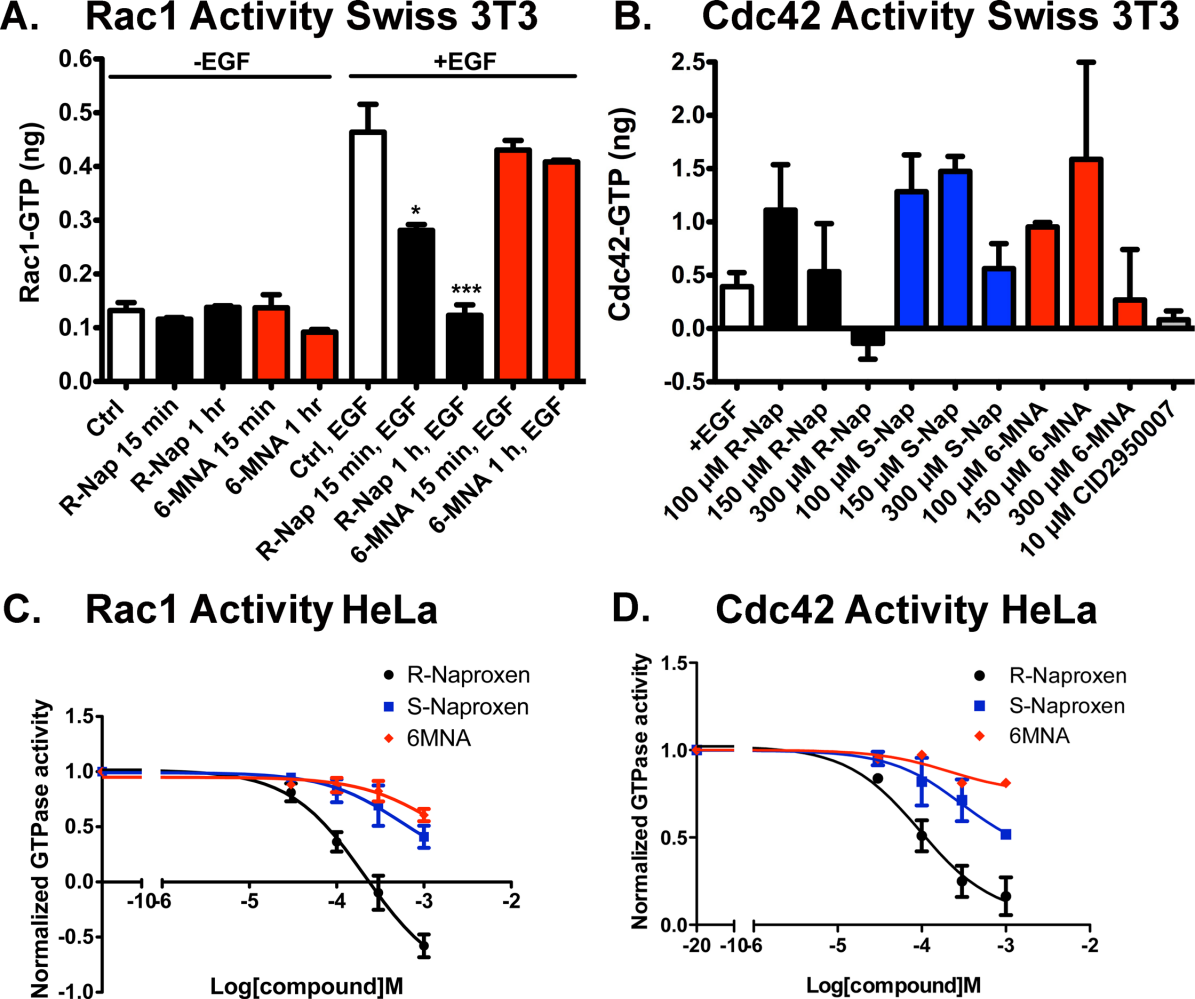
R-naproxen inhibits Rac and Cdc42 activation in response to growth factor stimulus of cells. (**A-B**) GLISA effector binding assays were used to quantify Rac1 and Cdc42 GTPase activities in Swiss 3T3 fibroblast cell lysates following preincubation for varying times (15 min or 1 h) or with varying concentrations of R-naproxen, S-naproxen or 6MNA, with or without (+/-) 100 ng/ml EGF stimulation. In panel A, R-naproxen and 6-MNA were used at 300 μM and time of exposure varied as indicated. One-way ANOVA and Dunnett’s multiple comparison test shows select R-Naproxen samples significantly (*p<0.05, ***p<0.001) different from EGF stimulated controls. In panel B, drug doses ranged from 10–300 μM as indicated. CID2950007 is a selective inhibitor of Cdc42 and served as a positive control. N = 3–8. One-way ANOVA and Dunnett’s multiple comparison test did not identify significant differences relative to EGF-stimulated controls. (**C-D**) Flow cytometric G-trap assay was used to quantitatively assess dose dependent inhibition of Rac1 and Cdc42 activation in HeLa cells following 2 h pre-treatment with R-naproxen, S-naproxen or 6MNA (30–1000 μM) and 2 min EGF stimulation (100 ng/ml). The inhibition curves were fitted to the sigmoidal dose-response equation in GraphPad Prism 5. Quantification of three independent measurements are plotted ± SEM.

Because R-naproxen displayed a significant difference as compared to S-naproxen and 6-MNA in its *in vitro* activity against GTPases (Fig 1, Figure B in S1 File), we examined R-naproxen, S-naproxen and 6-MNA effects in some detail for modulation of Rac1 and Cdc42 activity in cells. Small G-protein activation assays (GLISA) provide a method for measuring the activation status of Rac1 or Cdc42 in living cells (Fig 2). EGF increases Rac1 activity 5- to 10-fold above basal levels with maximal activity evident at 2 min post-stimulation in Swiss 3T3 fibroblasts and HeLa cells. Using GLISA, 300 μM R-naproxen pretreatment of Swiss 3T3 cells for 15 min significantly inhibited EGF-mediated Rac1 activation relative to EGF stimulated controls and a 1 h pretreatment reduced activation to basal unstimulated levels (Fig 2A). 6-MNA had no inhibitory effect at either time point. R-naproxen pretreatment of Swiss 3T3 cells for 1 h also showed a trend of dose-dependent inhibition of EGF-stimulated Cdc42 activation, which was maximal at 300 μM drug and comparable to treatment with 10 μM of Cdc42 specific inhibitor CID2950007/ML141 (Fig 2B). Unlike Rac1 activity, the relatively low levels of Cdc42 activity in Swiss 3T3 cells were more difficult to measure reproducibly via GLISA, and despite many trials the data did not reach statistical significance. Independent dose response measurements were made possible by our development of a G-trap effector binding assay that provides a highly quantitative flow cytometric read-out and a greater dynamic range [55].

HeLa cells were used as an independent cancer-relevant, human cell line to test the full chemical series (R-naproxen, S-naproxen, and 6-MNA) for effect on Rac1 and Cdc42 activation using the G-trap assay (Fig 2C and 2D). The G-trap assay gave highly reproducible dose response curves with each data point ± SEM representing triplicate samples measured in three independent trials. R-naproxen selectively inhibited Rac1 activity in a dose dependent manner with an EC_50_ of 212 μM; while S-naproxen and 6MNA had minimal inhibitory effect at this dose and up to 6-fold lower potency (Fig 2C). Maximal inhibition of EGF stimulated activity was observed at 300 μM and inhibition by R-naproxen below baseline indicates inhibition of non-stimulated basal activity at doses above 300 μM; suggesting there may be cell type specific differences in potency and efficacy. R-naproxen also selectively inhibited Cdc42 activity in a dose dependent manner with an EC_50_ of 96 μM; while S-naproxen and 6MNA had little inhibitory effect at this dose and 3-fold lower potency overall (Fig 2D).

Differential cytotoxicity of R-naproxen as compared to S-naproxen was ruled out using an LDH release assay (Figure C in S1 File). R-naproxen, but not S-naproxen or 6-MNA, had a modest inhibitory effect on cell proliferation in two different ovarian cancer cell lines relative to mock treated cells with extended treatment (Figure C in S1 File). The inhibition of proliferation by R-naproxen was similar to that observed using the Rac1 inhibitor, NSC23766 (Figure C in S1 File).

**Fig 3:**
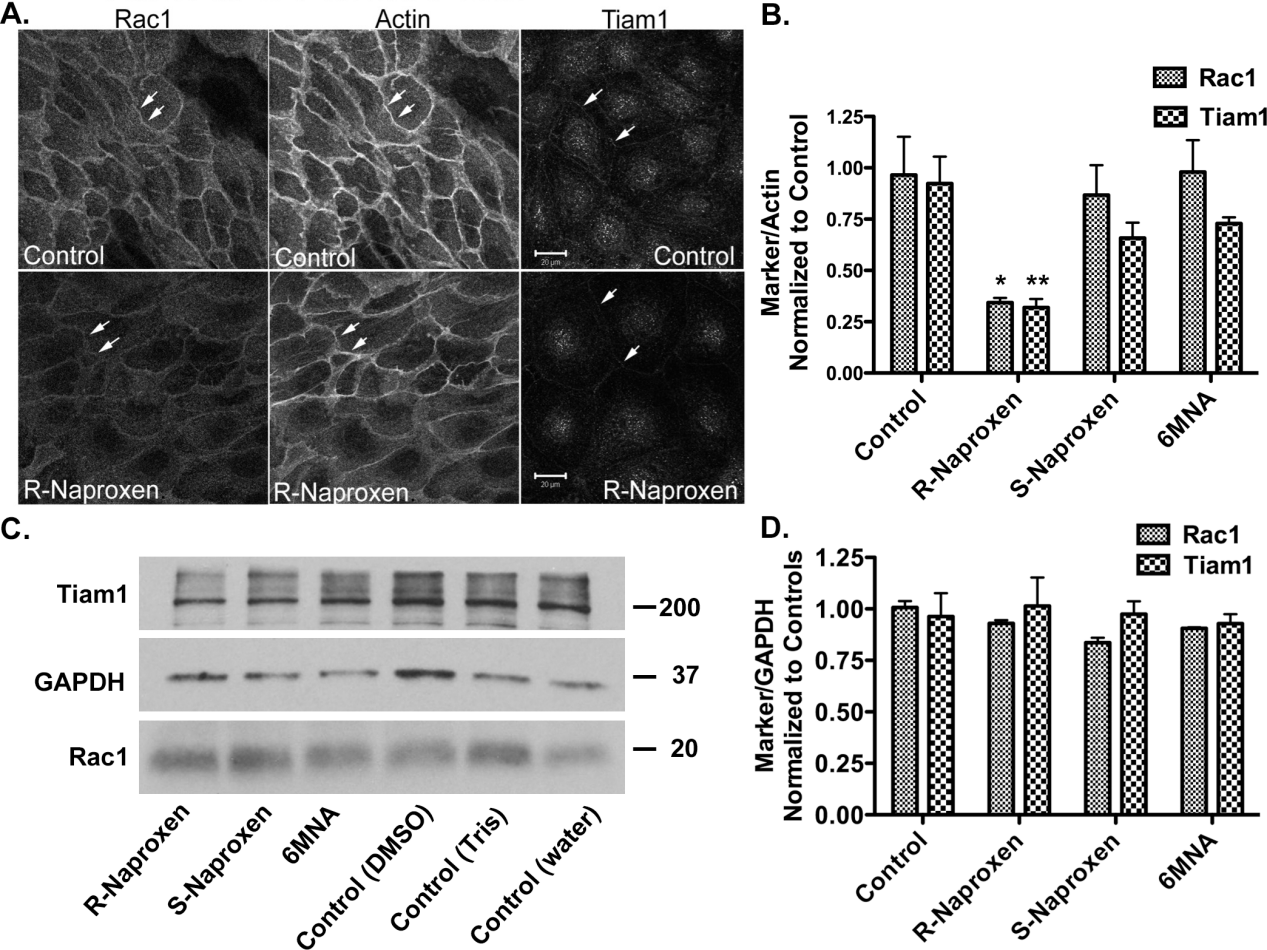
R-naproxen alters membrane localization of Rac1 and TIAM1 GEF. **(A)** OvCa433 cells were left untreated or treated with R-naproxen (300 μM) for 24 h and subsequently fixed and multiply stained for Rac1, actin (phalloidin) and Tiam1. N = 3. See also Figure D in S1 File for time dependent loss of Rac1 membrane localization. N = 3 (**B**) Quantification of image intensity using Image J of 3 representative confocal images as shown in panel (A) containing 20–30 cells/field normalized to actin and untreated controls. (**C**) Western blot analyses of lysates from cells treated with 300 μM compound as indicated for 24 h as compared to untreated controls. 6-MNA (30 mM stock in DMSO), S-naproxen (30 mM stock in water), or R-naproxen (30 mM stock in 1 M Tris buffer, pH 8). Blots were probed for Tiam1 (200 kDa), GAPDH (37 kDa), Rac1 (20 kDa), as a loading control. (**D**) Quantification of image intensity using Image J. Each marker was normalized to GAPDH and untreated controls. N = 3. One-way ANOVA and Dunnett’s multiple comparison test shows select R-Naproxen samples significantly (*p<0.05, **p<0.01) different from controls.

Membrane localization of Rac1 and Cdc42 are important indicators of GTPase activation. Tiam1 is a guanine nucleotide exchange factor (GEF) responsible for Rac1 activation and co-localizes with active Rac1 at the plasma membrane and in membrane ruffles. The membrane localizations of Rac1 and Tiam1 are both substantially reduced in the presence of R-naproxen in a time dependent manner (Fig 3A and 3B and Figure D in S1 File). S-naproxen and 6-MNA had no effect on Rac1 localization and only slightly reduced Tiam1 membrane staining (Fig 3B and Figure D in S1 File) consistent with the inhibition of Rac1 activity measured by GLISA (Fig 2). The naproxen series of compounds (R-naproxen, S-naproxen and 6-MNA) did not alter total Rac1 or Tiam1 protein levels measured by immunoblot (Fig 3C and 3D). *In vitro* assays measuring the effect of R-naproxen on GEF activities for Rac1 and Cdc42 specific GEFs did not reveal any inhibitory effects (Figure E in S1 File). The impact on Rac1 localization was selective; aspirin, ibuprofen, fenoprofen and ketoprofen did not alter membrane localization of Rac1 (not shown). These findings confirm the R-enantiomer-selective actions of naproxen within live cells.

### Enantiomer-selective inhibition of Rac1 and Cdc42 dependent cell migration and cytoskeletal rearrangements

**Fig 4:**
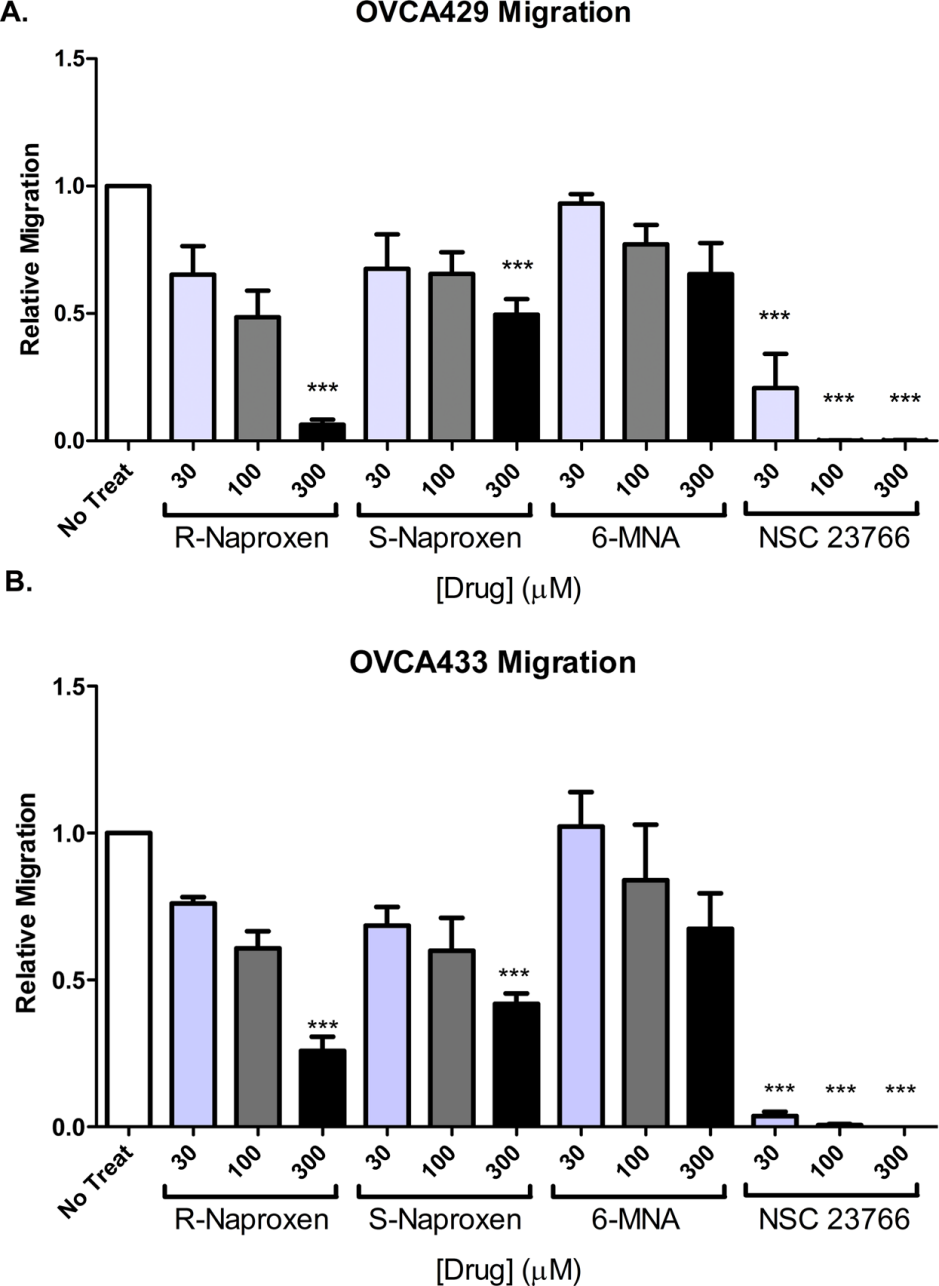
R-naproxen inhibits migration of immortalized human ovarian cancer cells. Cell migration was measured using a Boyden chamber assay. (**A-B**) Ovarian cancer (OvCa429 and OvCa433) cells were plated on filters and treated with 0–300 μM R-naproxen, S-naproxen, 6-MNA or the Rac1 inhibitor NSC23766 and then allowed to migrate for 48 h. Migrated cells were photographed and counted in three independent fields per chamber. N = 3 One-way ANOVA and Tukey's multiple comparison test shows values significantly (**P<0.01, ***P<0.001) different from untreated controls.

Cell morphologic changes required for migratory responses and invasive behaviors are known to be highly dependent on active Rac1 and Cdc42 GTPases. Comparative analyses of the effects of R-naproxen, S-naproxen and 6 MNA on cell migration showed R-naproxen to have a statistically significant inhibitory effect at 300 μM in two ovarian cancer cell line (OvCa429 and OvCa433) (Fig 4A and 4B) compared to untreated controls.

**Fig 5:**
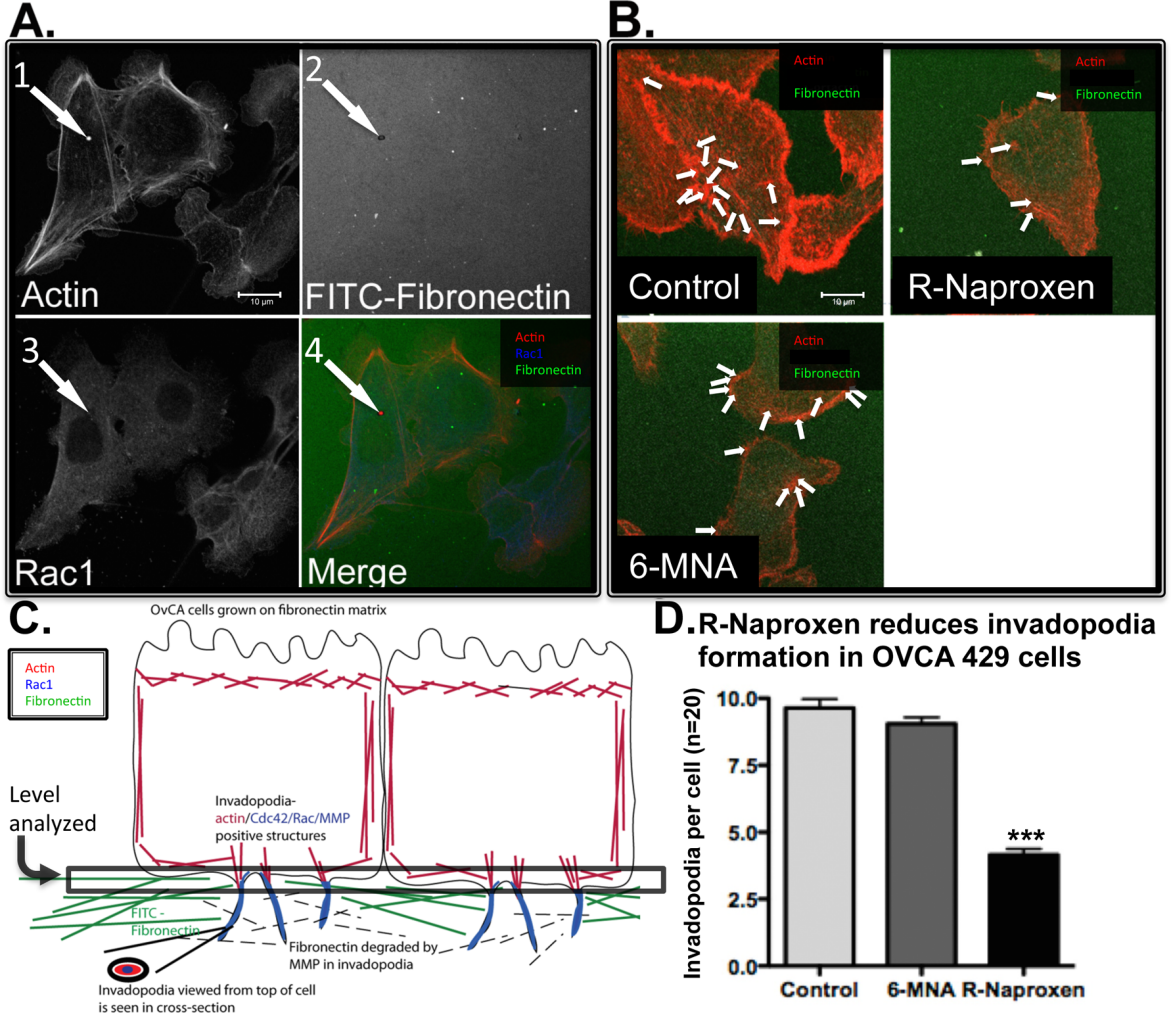
R-naproxen selectively inhibits invadopodia formation. (A) Arrowheads indicate the three defining features of each invadopodia quantified: 1. Puncta of actin; 2. Degradation of FITC-fibronectin; 3. Puncta of Rac1 or cortactin; 4. Merge channels to ensure three factors convalesce. (B) Cells plated on FITC-fibronectin coated substrates were cultured with R-naproxen, 6 MNA, or left untreated, then EGF stimulated, and invadopodia quantified. Representative cells from each treatment condition are shown, with invadopodia quantified indicated by arrowheads. (C) Schematic of invadopodia assay. (D) Invadopodia were quantified based on local degradation of fibronectin substrate, and costaining with cortactin (not shown). N = 3, with N = 20 cells quantified per treatment condition. One-way ANOVA and Dunnett’s multiple comparison test shows R-Naproxen samples significantly (**p<0.001) different from control and 6MNA.

Activated GTPase induced actin rearrangements are key to cell morphologic changes in response to growth factor stimulus and serve to regulate cell migration on and adhesion to substrate. Analyses of cell morphology on fibronectin coated surfaces with and without R-naproxen slowed growth factor induced cell morphology changes and most significantly cell rounding and loss of substrate adhesion (Figure F in S1 File). Of particular interest for cancer metastasis, Cdc42-dependent, actin-based invadopodia are crucial for matrix degradation and invasion. Invadopodia formation in the presence of R-naproxen was inhibited relative to the inert 6-MNA derivative (Fig 5).

**Fig 6:**
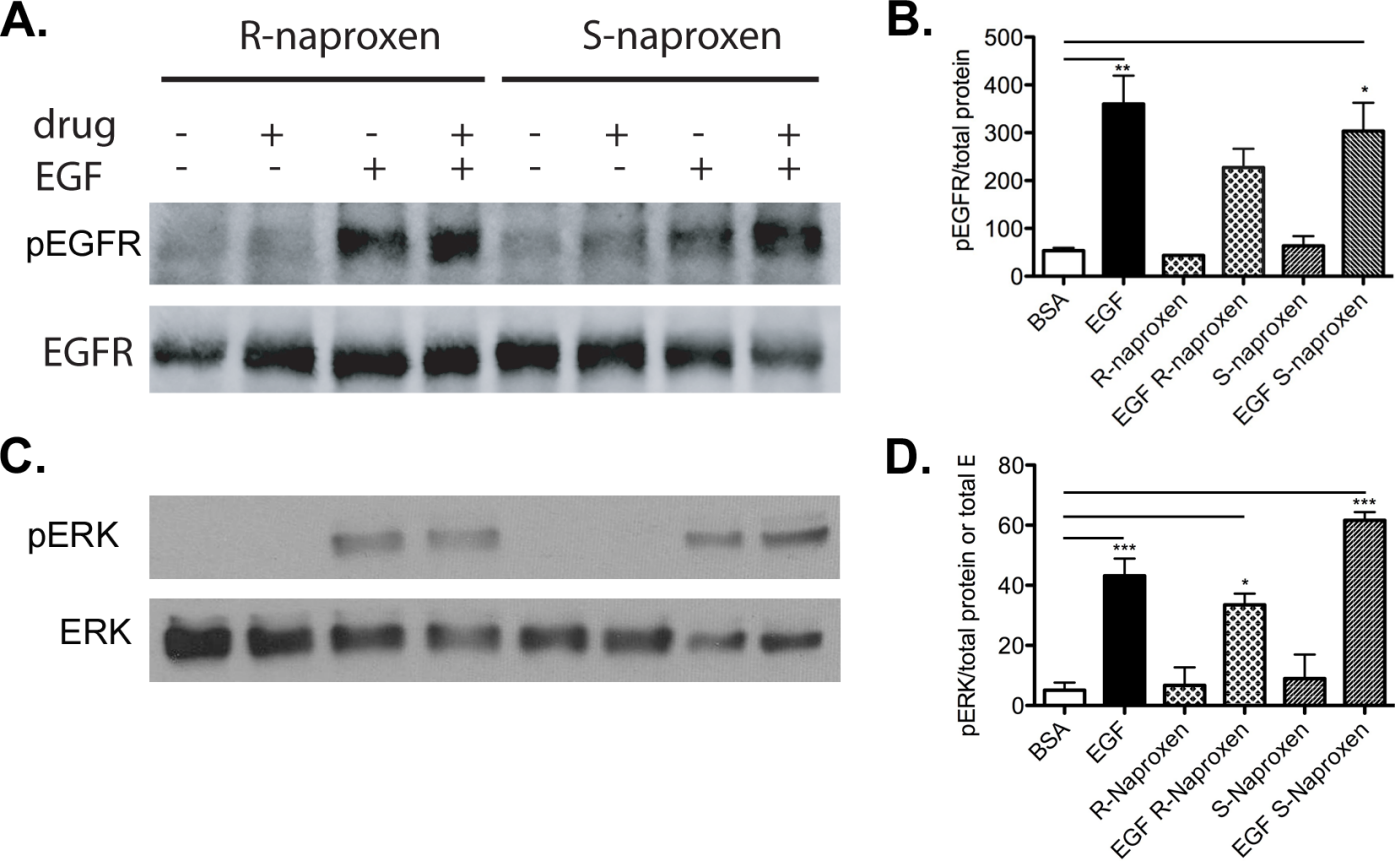
R-naproxen acts via a COX-independent mechanism. OvCa433 cells were left untreated (BSA only) or incubated with or without 300 μM of S-naproxen or R-naproxen for 48 h. Thereafter, stimulation was for 10 min with EGF where indicated. Cell lysates were resolved by SDS-PAGE and immunoblotted for phosphorylated EGFR (pEGFR) or phosphorylated ERK (pERK). Phosphorylation of EGFR and ERK were determined in the presence of S-naproxen (SN) or R-naproxen (RN), without (BSA) or with EGF (EGF). Shown are western blots probed with phospho-specific antibodies for (**A**) pEGFR and (**C**) pERK relative to immunoblots for total EGFR or ERK proteins. Bar graphs show quantification of each phosphoprotein by densitometry and normalized to total protein (measured either by immunoblot or via Coomassie staining) (**B**) pEGFR/total protein and (**D**) pERK/total protein or total ERK. N = 2 One-way ANOVA and Tukey’s multiple comparison test shows EGF-stimulated samples +/- drug values significantly (*p<0.05, **p<0.01, ***p<0.001) different from BSA controls as indicated on the graph. Unstimulated samples +/- drug were not statistically different when compared pairwise and the same was true for pairwise comparisons of stimulated samples +/- drug.

Both COX-1 and COX-2 are implicated in ovarian cancer pathogenesis [87, 88]. Ovarian cancer cells harbor COX-1 and COX-2 enzymes and the main metabolic intermediary, COX-2, can regulate cell migration via the intracellular activation of EGFR [89]. Therefore, the possibility that R-naproxen inhibits cell migration through a COX-dependent mechanism involving a reduction in PGE2 was tested with the expectation that the NSAIDs would cause a decrease in EGFR phosphorylation and downstream targets. Contrary to the expectation for a COX/PGE2-mediated mechanism, human ovarian cancer (OvCa433) cells incubated with 300 μM of S-naproxen or R-naproxen +/-EGF exhibited levels of phosphorylated EGFR (pEGFR) and (pERK) that were statistically above unstimulated controls (BSA, p values on graphs) and similar to controls treated with EGF alone (without naproxen) (Fig 6A–6D). These results demonstrate that 1) R-naproxen is not acting by blocking COX-mediated activation of the EGFR pathway and 2) there is no significant enantiomer selective difference in EGFR activation that can account for the observed differential effects on migration. For control purposes, the effect of R-naproxen on Rac1 GTPase activation in OvCa433 cells was tested in parallel and confirmed to have a similar inhibitory effect to that documented in 3T3 and HeLa cells (Figure G in S1 File). Therefore, it is likely that R-naproxen is reducing migration by a mechanism independent of COX inhibition.

### Cheminformatic analysis predicts enantiomer selective interaction of R-naproxen and R-ketorolac with Rac1 and Cdc42

**Fig 7:**
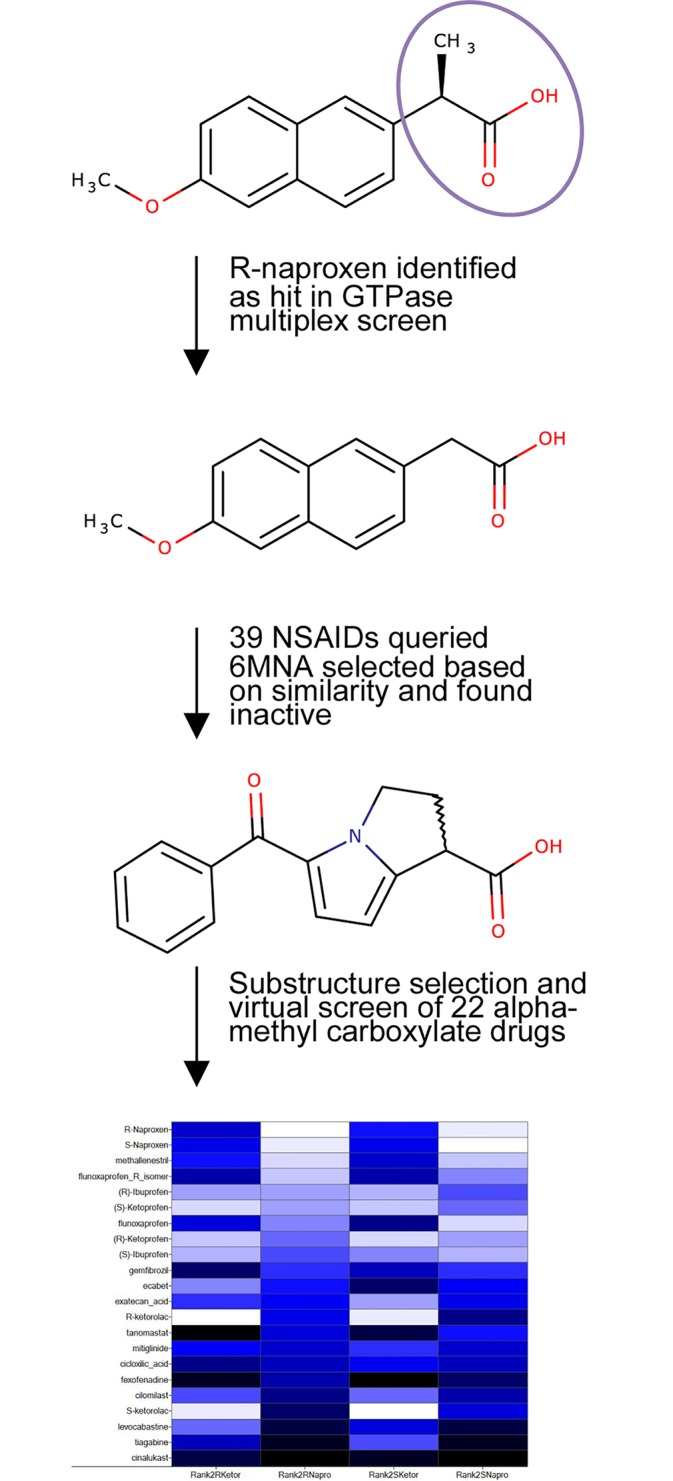
Schema of structure activity evaluation and substructure selection for virtual screen. Differential activities in cell based studies of the chemically related structural series encompassing R-naproxen, S-naproxen and 6-MNA prompted focus on the α-methyl carboxylate as a critical structural determinant. With R-naproxen as a query and focusing on α-methyl carboxylates, we evaluated all approved NSAIDs and α-Me-COOH drugs (“rotational barrier” hypothesis). In total 39 NSAIDs, including 15 α-Me-COOH launched drugs, were evaluated; ketorolac (as separate enantiomers) was on the list, and the racemic mixture was selected based on its approval for human use. See Figure G in S1 File for detailed heatmap that illustrates the less-than-perfect overlap of the two queries, R-naproxen and R-ketorolac due to the significant differences in their structures.

The selective inhibitory activity of R-naproxen against Rac1 and Cdc42 while intriguing, was countered by the facts that: 1) only S-naproxen is approved for human use (Aleve or Naprosyn) and 2) relatively high concentrations are required for biological effect even if still within the clinically indicated dosages and serum concentrations [61, 62]. The limitations of R-naproxen for clinical translation motivated cheminformatics analyses and a virtual screen to identify an approved drug for clinical use that retained stereoselectivity. Since S-naproxen and 6-MNA lacked Rac1 and Cdc42 activity (Fig 1), we postulated that rotational constraints around the chiral center imposed by the methyl group and a requirement for the aryl rings to be accommodated in a hydrophobic pocket might explain enantioselective GTPase inhibition (Fig 7). Similarity queries using R-naproxen as the query, focused on 22 drugs with rotationally constrained α-methyl carboxylate chiral centers, as well as di-methyl carboxylates (see Methods and Tables D and E in S1 File for further details). Among these compounds R- and S-ketorolac were notable because the two enantiomers were ranked differentially in activity by the query (Figure H in S1 File). Although a reverse query using R-ketorolac resulted in less than perfect overlap with the R-naproxen query, we were encouraged by the fact that there was good congruence among compounds identified as actives in the initial screen, e.g. ibuprofen and ketoprofen (Table A in S1 File). Because ketorolac was not present in the Prestwick Chemical Library^®^ that was screened and because its S-enantiomer has higher biological efficacy against COX enzymes, both R- and S-ketorolac were selected for further testing. An additional argument was the fact that R/S ketorolac (as racemic mixture) is approved for human and can be actively pursued for clinical testing (ClinicalTrials.gov, NCT01670799) [90].

### Enantiomer selective inhibition of Rac1 and Cdc42 by R-ketorolac

**Fig 8:**
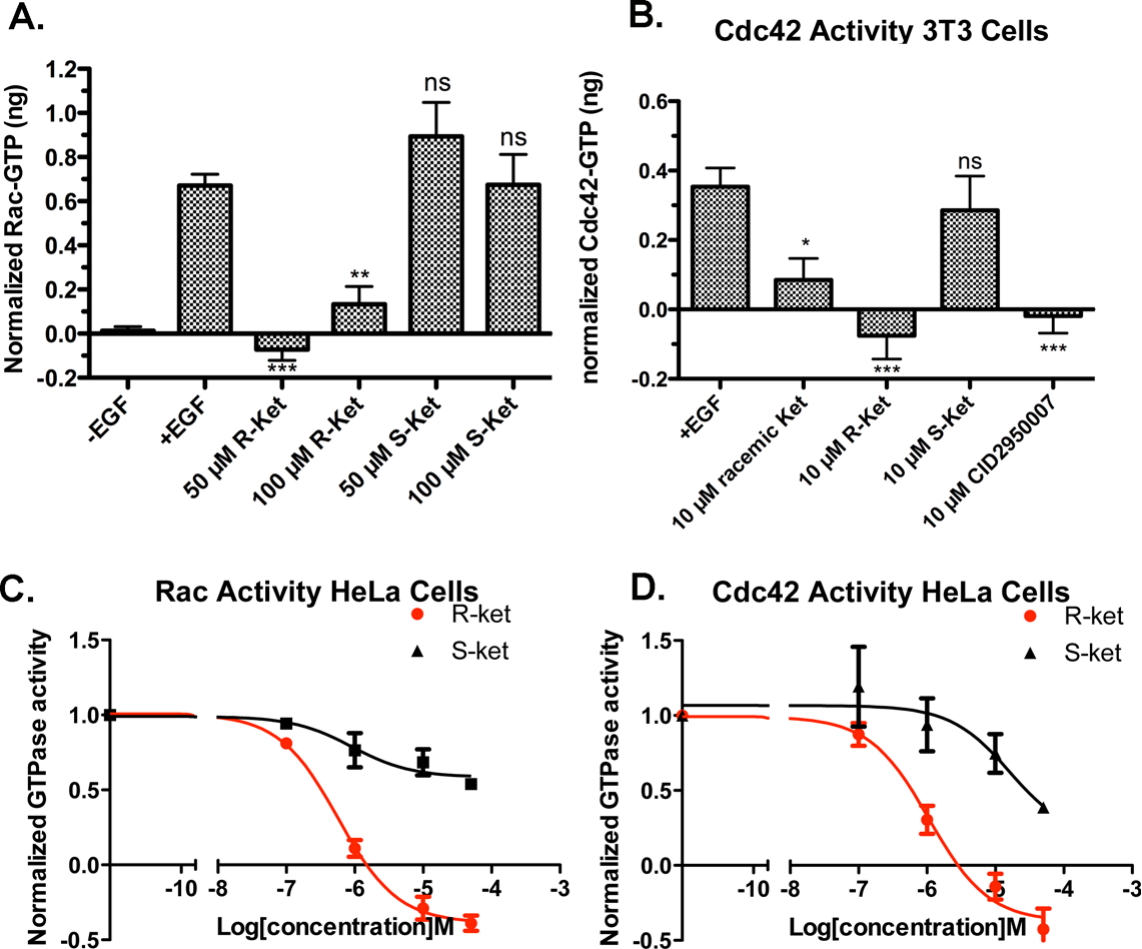
Ketorolac exhibits enantiomer selective inhibitory properties against Rac1 and Cdc42. **(A-B)** GLISA effector binding assays were used to quantify Rac1 and Cdc42 GTPase activities in cell lysates (Swiss 3T3 cells) following 1 h preincubation with varying concentrations of R- and S-ketorolac with and without EGF stimulation. N = 3 or more trials. One-way ANOVA and Dunnett’s multiple comparison tests shows R-ketorolac samples and CID2950007 (Cdc42 inhibitor) significantly different (*p<0.05, **p<0.01, ***p<0.001) from EGF stimulated controls. (**C-D**) Flow cytometric G-trap effector binding assays were used to determine dose dependent inhibition by R-ketorolac vs. S-ketorolac in HeLa cells. R-ketorolac EC_50_ against Rac1 = 0.574 μM; R-ketorolac EC_50_ against Cdc42 = 1.07 μM. The inhibition curves were fitted to the sigmoidal dose-response equation in GraphPad Prism 5. Quantification of three independent measurements are plotted ± SEM.

The virtual screening prediction for the selectivity of ketorolac enantiomers against Rac1 and Cdc42 GTPases was tested in multiple cell-based assays. Initially, an ELISA-type, effector-binding assay (GLISA, Cytoskeleton Inc.) was used for measuring the pool of active GTPases in cells +/- treatment with R-, S- and racemic ketorolac as compared to known GTPase selective inhibitors not approved for human use. In Swiss 3T3 cells, R-ketorolac exhibited maximal inhibitory activity against both Rac1 and Cdc42 at doses of 50 and 10 μM, respectively, as compared to S-ketorolac, which was non-inhibitory at these doses (Fig 8A and 8B). R-ketorolac inhibitory activity against Cdc42 was similar to that of a Cdc42 specific inhibitor with μM potency, while racemic ketorolac at 10 μM exhibited approximately half the effect at the same dose. S-ketorolac was non-inhibitory (Fig 8B). We performed dose-response measurements with a novel, highly sensitive flow cytometry assay for more quantitative analysis and suited for EC_50_ determination [55]. In brief, GST-chimeras of GTPase effector binding domains are coated on 10 μm glutathione beads that are suitable for flow cytometric analysis. Detergent cell lysates are prepared under conditions that minimize GTP hydrolysis and small amounts of cell lysate protein are immediately incubated on ice with the effector coupled beads for 1 h. Active, GTP-bound GTPases are subsequently quantified using specific, fluorescently labeled antibodies and flow analyses. Negative controls include beads coated with an irrelevant effector, and non-specific, isotype matched antibodies. Using a G-trap effector binding assay to monitor cellular effects in a second cell line (HeLa), R-ketorolac exhibited significant preferential inhibitory activity against Rac1 and Cdc42 with EC_50_ values of 0.574 μM and 1.07 μM, respectively (Fig 8C and 8D). S-ketorolac failed to reach a threshold of 50% efficacy against Rac1 and was virtually inactive against Cdc42 with an estimated EC_50_ of >10 μM based on the data shown. Table F in S1 File provides a summary of serum concentrations, effective doses of NSAIDs as compared to selective Rac and Cdc42 inhibitors.

### Docking studies suggest enantiomer selective interaction of R-naproxen and R-ketorolac with Rac1 and Cdc42

**Fig 9:**
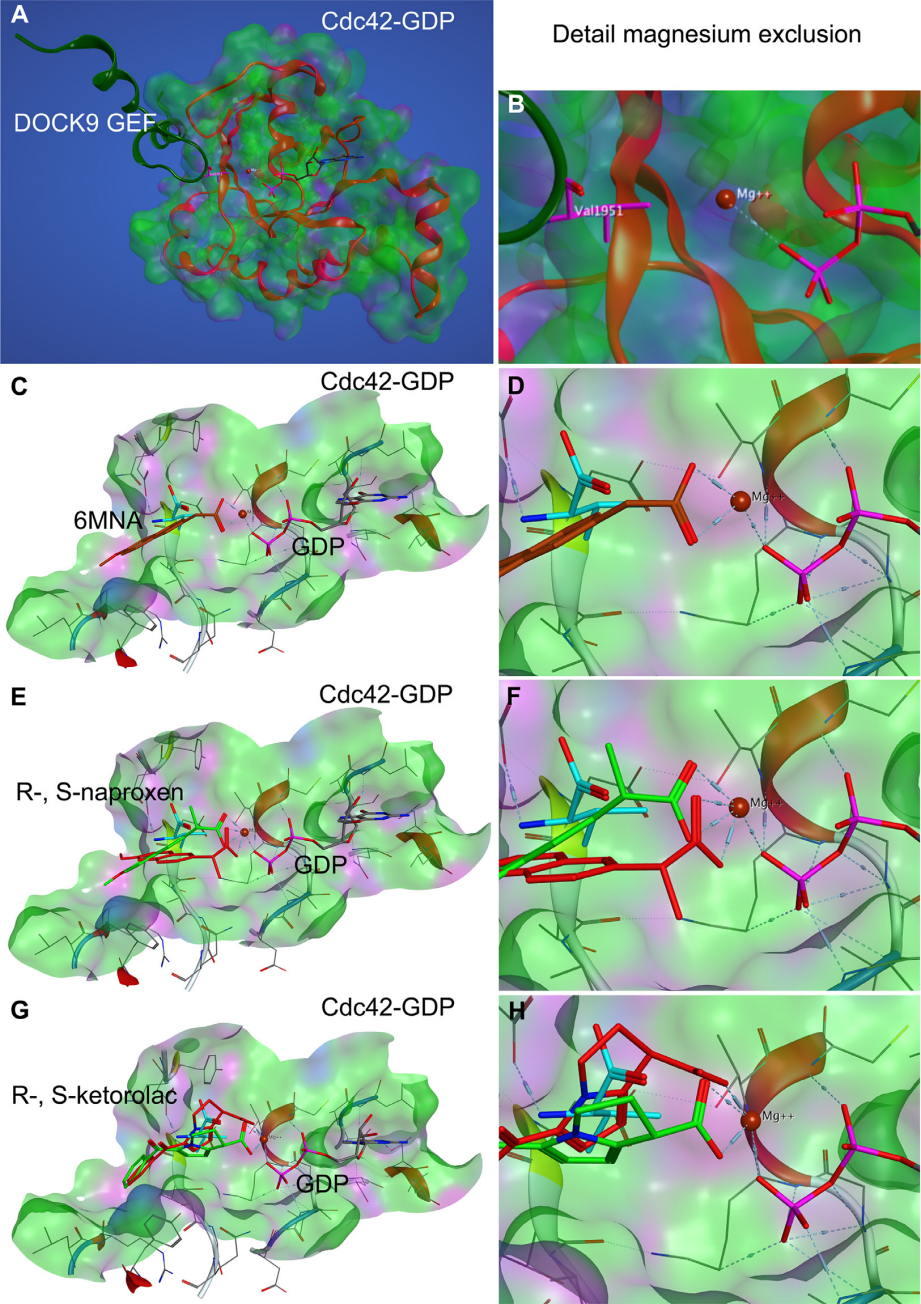
Compound docking to Cdc42 based on DOCK9 magnesium exclusion model. The docking model is based on the work of Yang et al., 2009 [77] wherein Val1951 in the sensing domain of the DOCK9 GEF (dark green ribbon) was suggested to reduce nucleotide interaction with Mg^2+^ via steric hinderance or ‘exclusion’ and thereby destabilize nucleotide binding to cause release from the GTPase active site. **(A-B)** The crystal structure of Cdc42-GDP in complex with the DOCK9 GEF (PDB ID 2WMN) was used to predict the active site docking of (**C-D**) 6-MNA, (**E-F**) R-, S-naproxen and (**G-H**) R-, S-ketorolac. (**B, D, F, H**) The carboxyl moiety in all compounds is proposed to interact with the Mg^2+^, thereby reducing interaction with GDP and reducing binding affinity analogous to Val1951 (teal). R-naproxen and R-ketorolac are shown in red. 6-MNA rust, S-naproxen and S-ketorolac are shown in green. R-enantiomers show more favorable interaction with Mg^2+^ than S-enantiomers due to rotational constraints imposed on the carboxylate by the stereocenter. For quantification of free energy of ligand binding and distances see Table G in S1 File.

**Fig 10:**
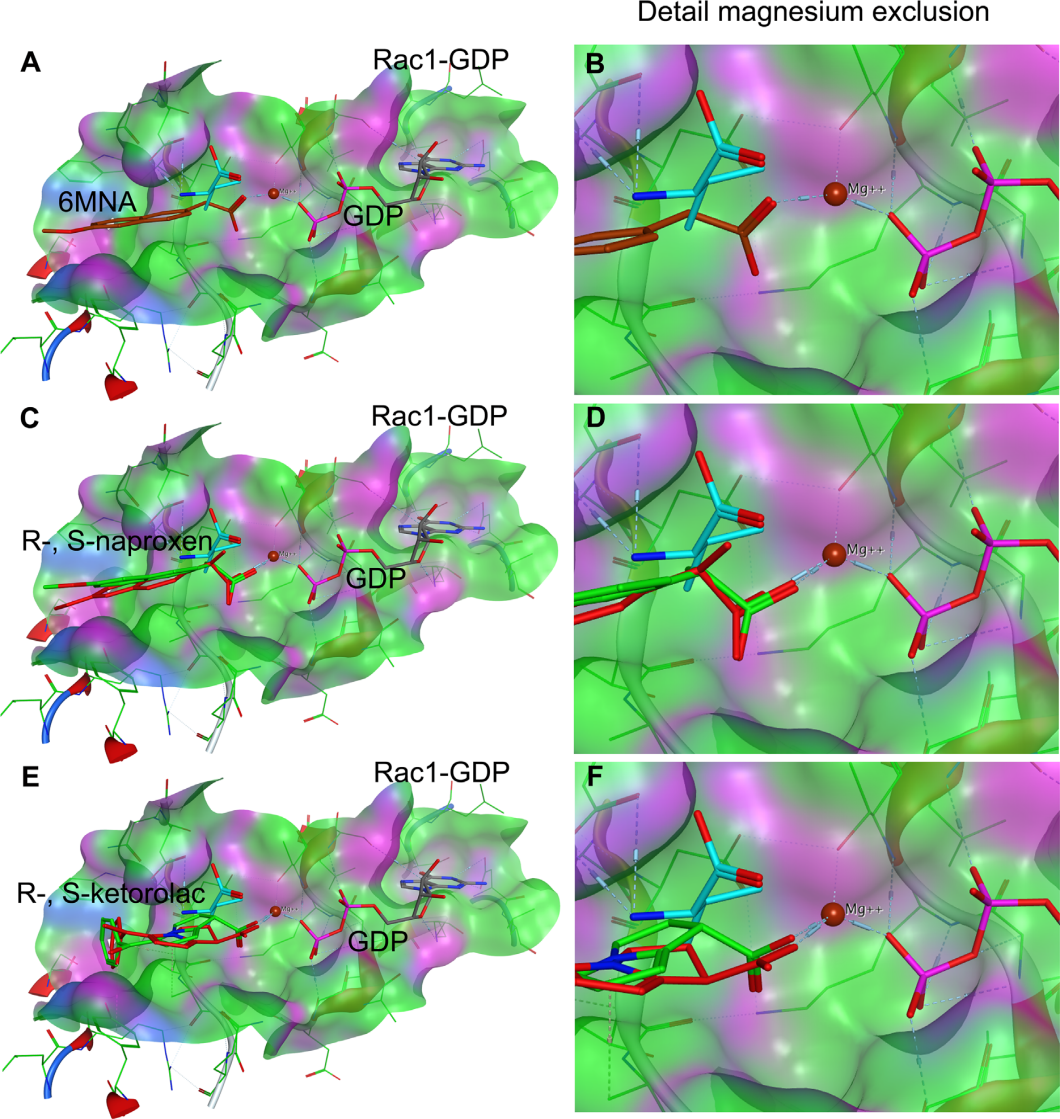
Compound docking to Rac1. NSAID docking on Rac1 was modeled on the ‘magnesium exclusion’ model proposed based on the crystal structure of Cdc42 complexed to DOCK9 GEF [77] and subsequently also validated for the crystal structure of Rac1 complexed to DOCK2 [78]. **(A-B)** The crystal structure of Rac1-GDP in complex with the DOCK9 GEF (PDB ID 2YIN) was used to predict the active site docking of 6-MNA, (**C-D**) R-, S-naproxen and (**E-F**) R-, S-ketorolac. The carboxyl moiety in all compounds is proposed to interact with the Mg^2+^, thereby reducing interaction with GDP and binding affinity analogous to Val1539 (teal) in the DOCK2 GEF (see Fig 9 for detail). R-naproxen and R-ketorolac are shown in red. 6-MNA rust, S-naproxen and S-ketorolac are shown in green. R-enantiomers show more favorable interaction with Mg^2+^ than S-enantiomers due to rotational constraints imposed on the carboxylate by the stereocenter. For quantification of free energy of ligand binding and distances see Table G in S1 File.

Structural studies of guanine nucleotide exchange factor (GEF)-mediated nucleotide exchange on Cdc42 and Rac1 demonstrated the importance of a highly conserved valine residue in the nucleotide sensing domain of the DOCK9 (Val1951) and DOCK2 (Val1540* or Val1539**, note position differs in Kulkarni et al.*, as compared to crystal structure** and human DOCK2 amino acid sequence NP_004937.1**) GEFs [77, 78]. The insertion of a sensing domain valine occludes the critical coordination of magnesium by the nucleotide and the consequent reduced charge shielding decreases nucleotide affinity and leads to nucleotide release from the GTPase. The mechanism of magnesium exclusion to promote nucleotide release is distinct from that of other GEF proteins and is suggested to enhance DOCK GEF activity against a broader range of GTPases [77]. The observed importance of the NSAID alpha carboxylate group in their GTPase inhibitory activities prompted us to consider magnesium exclusion within the nucleotide binding pocket as a possible mechanism for destabilization of nucleotide binding by R-naproxen, R-ketorolac and 6-MNA through docking analyses. Other allosteric pockets on Rac1 and Cdc42 have been proposed as possible drug binding sites through a combination of comparisons of crystallographic data of various nucleotide bound states and molecular simulations [79]. Although the proposed allosteric binding pockets from simulation studies show great promise in terms of developing novel small molecule modulators the provided evidence of RhoA activation is indirect and insufficient to validate interaction of small molecule modulators with proposed allosteric pockets. Further work is required for validation by either structure elucidation or mutation studies. The binding mode proposed here is based on evidence from structural data on protein—protein interaction (GEF-GTPase) and we consider it to show similar promise until experimentally validated. Comparisons against the S-enantiomers of naproxen and ketorolac, along with an amide derivative were performed in parallel. The carboxyl moiety in all of the compounds has the potential to neutralize or displace Mg^2+^ thus reducing nucleotide binding affinity (Figs 9 and 10, Table G in S1 File). In 6-MNA, the lack of a methyl group to restrict free rotation of the carboxyl increases entropy and decreases free energy of binding compared to naproxen. The docking poses suggest that there may be some steric hindrances preventing S-enantiomers from forming a more stable coordination with Mg^2+^ (Table G in S1 File), thus leading to a more favorable interaction of R-enantiomers in congruence with the *in vitro* data. Ketorolac has even lower degrees of freedom compared to naproxen because the stereo center is conformationally restrained in a ring, which is likely to lead to more stable complex formation with Mg^2+^ and could explain the higher observed potency.

## Discussion

The benefit of NSAIDs as general chemopreventive and anti-cancer agents has been debated due to mixed epidemiologic evidence [91–98]. Recent renewed interest is based on retrospective efficacy studies of select NSAIDs and the demonstration that some NSAIDs have non-COX mediated effects on cancer relevant pathways [99–103]. Nevertheless the field is hampered by differences in findings based on tumor type, limited knowledge on response stratification based on specific NSAID use, and lack of information on potential enantiomer-dependent pharmacologic activities of certain NSAIDs. Here, we provide evidence that select R-enantiomers of NSAIDs (R-naproxen and R-ketorolac) inhibit the small GTPases Rac1 and Cdc42. In separate high throughput screens using different libraries, a specific NSAID (R-naproxen) or an NSAID-related compound (CID2950007/ML141) were identified as Rac1 and/or Cdc42 inhibitors. Among the identified Cdc42 selective inhibitors, a family of pyrazolines (exemplified by CID2950007/ML141) shows striking similarity to the COX-2 inhibitor celecoxib, yet celecoxib itself shows no activity against GTPases demonstrating that small differences in structure can dramatically affect activity [30, 80]. Reports that Ras signaling is a target of sulindac sulfide offers a further example of NSAID effectiveness against GTPase pathways in cancer [36, 37]. The composite data demonstrate that non-COX targets may play important roles in the anti-cancer activities of individual NSAIDs.

Enantiomeric drug pairs have long been recognized to differ greatly in biologic response [104] with thalidomide serving as a recent dramatic example [105]. Many NSAIDs are FDA-approved and administered as a 1:1 racemic mix of the R- and S-enantiomers with the S-forms displaying inhibitory activity toward cyclooxygenase (COX) enzymes [38, 43, 44, 106–108], naproxen being one of the few exceptions that is available for human use as the S-enantiomer only. Despite many examples of enantiomer-selective drug targets [11], little is known about the bioactivities of NSAID R-enantiomers. Although the R-forms have little activity against COXs [38, 43, 44, 106–108], there is mounting evidence that R-enantiomers are distinct chemical entities and precedence for pharmacologic activities dictated by R-enantiomers of specific NSAIDs against novel (non-COX) targets [47–49, 109–111]. In the context of cancer, R-etodolac and subsequent analogs SDX-301 and SDX-308 display anti-tumor activity in chronic lymphocytic leukemia and activity against multiple myeloma in cell and animal models [111–115]. R-etodolac also significantly suppressed tumors in a colitis-related mouse model of colon cancer [47] and retarded tumor development and metastasis in a transgenic mouse model of prostate cancer [110]. The target of R-etodolac has not been definitively established; the retinoid X receptor-α was identified as the target in prostate cancer and R-etodolac is associated with inhibition of the Wnt beta-catenin/TCF signaling pathways [116] in hematologic malignancies.

The HTS confirmed hit R-naproxen, identified from the Prestwick Chemical Library^®^, as well as the rationally selected enantiomer, R-ketorolac, substantiate the hypothesis that R-enantiomers of NSAIDs have a pharmacological profile that is different from their optical isomer counterparts. We observed such activities against Rho-family GTPases Rac1 and Cdc42 in particular. Data described in this report confirms the α-Me-COOH hypothesis and docking suggests that through coordination of the magnesium ion the carboxylate affects GTPase nucleotide binding affinity. Given that Mg^2+^ increases the affinity of nucleotide binding to GTPases, one can rationalize that the co-ordination of Mg^2+^ by ketorolac decreases the affinity. In the absence of Mg^2+^, we envision that ketorolac binding to an allosteric site near the nucleotide binding site can increase nucleotide binding affinity, but not to the extent of Mg^2+^ alone. Moreover, we would expect that the precise results would be sensitive to experimental conditions such as the local concentration of Mg^2+^, nucleotide, and drug on microspheres of different sizes and differing surface densities of GTPases; which may account for the observed behavior of R-naproxen as a stimulator or an inhibitor under different assay conditions. Preliminary solution-based, capillary thermophoresis measurements yielded K_D_ values for the direct interaction between Alexa-conjugated Cdc42 and ketorolac enantiomers that were consistent with the EC50 values measured by flow cytometry, though further follow-up will be required. Future structural studies will focus on further delineating the mechanism of action of these compounds.

For the Rho-family GTPases Rac1 and Cdc42, the modeled enantiomer-selective interaction predicts a novel mechanism of action based on stabilization of the GDP-bound GTPase that may in part be promoted through interference with GEF-mediated activation. The stabilization of the GDP-bound form as a potential mechanism of action is distinct from the currently known inhibitors. Farnesyl-transferase (FTI), geranylgeranyl-transferase (GGTI) and HMG-CoA-reductase inhibitors (i.e. statins) block GTPase membrane association. Current findings support Rho-family GTPases as viable therapeutic targets; however, isoprenoid pathway inhibitors display poor selectivity for individual Rho GTPases [22, 25, 117]. Other mechanisms are employed for selective inhibition of Rho-family GTPases, notably molecules to disrupt regulation of Rho-family GTPase activity by GEFs, GAPs and GDIs [29, 118, 119] or coupling with specific effectors [120]. The Rac1 inhibitor NSC23766 is an example of a compound that binds to the surface groove of Rac1 and disrupts GEF interaction [120]. Other inhibitors (e.g. Rac1 inhibitor EHT1864 or Cdc42 inhibitor ML 141/CID29950007) have been described that interfere with nucleotide binding [30, 120]. The identified Rac and Cdc42 selective inhibitors are effective in cell culture [28, 30, 34, 121, 122] and recent studies demonstrate anti-tumor activity in mouse xenograft models [32, 33], but these compounds have not been translated to human use. In contrast, the R-enantiomers of naproxen and ketorolac likely represent a new mechanism of action for Rac1 and Cdc42 inhibition and these chemicals show potential for rapid translation through drug repurposing approaches [35].

Recent retrospective and prospective analyses of peri-operative ketorolac administration indicates outcomes benefit in triple negative and high risk breast cancer patients [99–101, 123]. Our own retrospective analyses demonstrate a benefit of racemic ketorolac in ovarian cancer patients and R-ketorolac inhibits Rac1 and Cdc42 GTPases and tumor associated behaviors of human ovarian tumor cells both *in vivo* and *in vitro* [20, 124]. Taken together with the data presented here, it will be of interest to test the role of R-ketorolac in inhibiting Rac1 and Cdc42 GTPases in other solid tumor types (colon, breast, ovarian, lung, head and neck, prostate, renal) where these GTPases are increasingly recognized as important for adhesion, invasion and metastasis [32, 125–135]. The information provided by our analyses also offer a molecular mechanism for interpreting the clinical findings of trials in progress using ketorolac [90].

Racemic ketorolac is orally bioavailable, and its tromethamine salt is highly soluble and has been commercialized as topical (ocular, intranasal), injectable (intravenous or intramuscular), and oral formulations. This makes ketorolac an ideal candidate for human studies. Recently published studies of a Phase 0 trial in Stage III ovarian cancer patients offer proof-of-principle evidence for GTPase inhibition in patients treated with racemic ketorolac [20]. Furthermore, because R-ketorolac tends to bio-accumulate more than S-ketorolac in man [20, 108], the enantiopure drug R-ketorolac is posited to be an ideal candidate for new drug development.

## Supporting Information

S1 File **Figure A. R-naproxen selectively inhibits BODIPY-GTP binding by Rac1 and Cdc42 *in vivo*.** R-naproxen in contrast to S-naproxen and 6-methoxy naphthalene acid (6MNA) inhibited BODIPY-GTP binding to GSH bead immobilized GST-Rac1 and GST-Cdc42 GTPases in a dose-dependent manner. BODIPY-GTP concentration was held constant at 300 nM for these experiments and drug concentrations varied from 10 nM to 100 μM. Bead-associated fluorescence intensity was quantified by flow cytometry and used to monitor drug treatment induced changes in nucleotide binding. The inhibition curves were fitted to the sigmoidal dose-response equation using GraphPad Prism5. The EC_50_ of R-naproxen was 18 μM for both Rac1 and Cdc42, and an efficacy of 32% and 38%, respectively. The EC_50_ of S-naproxen was >200 μM for Rac1 and 4.2 μM for Cdc42 and the efficacy was 0% and 23%, respectively. 6MNA was inactive. N = 2. **Figure B. NSAIDs without effect on guanine nucleotide binding.** Dose response assays were conducted on eight Ras-related GTPases simultaneously using a multiplex format. **Figure C. R-naproxen is not cytotoxic or anti-proliferative.** (A-B) Ovarian cancer cells (OvCa433) were incubated for 48 h with R-naproxen or S-naproxen (0–300 μM) and lactate dehydrogenase (LDH) release into the media was determined by reduction of the tetrazolium salt INT into formazan relative to a positive control where membranes were permeabilized. (C-D) Effects on cell viability were measured by plating ovarian cancer cells (OvCa433) in 96-well plates in 10% FBS/MEM media and treating with R-naproxen or S-naproxen (0–300 μM) for 24–72 h. Viable cells were quantified using an MTS assay. (E) Proliferation of ovarian cancer cells (OvCa429 or OvCa433) treated with 0–300 μM each of R-naproxen, S-naproxen, 6 MNA or the Rac1 inhibitor, NSC23766 for a total of 6 days was measured using a BrdU assay. The results are from 3 independent experiments conducted in triplicate. **Figure D. R-naproxen selectively causes the time dependent dissociation of Rac1 from the plasma membrane.** Ovarian cancer cells (OvCa433) were left untreated or treated with 300 μM R-naproxen, S-naproxen or 6MNA for 1 h or 24 h prior to paraformaldehyde fixation. Samples were labeled for actin using rhodamine phalloidin and immunostained for Rac1 (mAb from BD Transduction Labs 610650 see main text for details). Rac1 staining was lost from the cell borders and seen accumulated in the perinuclear region at 24 h in R-naproxen treated samples, but not in S-naproxen, 6 MNA or control samples. Images were acquired on a Zeiss Axioskop outfitted with a digital camera. **Figure E. R-naproxen does not inhibit *in vivo* GEF activity.** (A) Real time measurement of mant-GTP binding in solution using purified Cdc42 and Dbs GEF domain with (blue squares) or without (black circles) 150 μM R-naproxen added. Plotted are data with control Cdc42 only values subtracted. Arrow denotes addition of hDBS GEF. (B) Real time measurement of mant-GTP binding in solution using purified Rac1 and PIP2/Tiam1 GEF. Plotted are fluorescence intensity measurements with DMSO or Rac1 only control values subtracted and then normalized to maximum fluorescence intensity. Curve fitting was performed using GraphPad Prism one site binding function. Curve fitting was performed using Prism. **Figure F. R-naproxen reduces growth factor dependent cell morphologic changes necessary for cell migration and adhesion.** (A) OvCa433 cells were plated on FITC- fibronectin and either left unstimulated or were stimulated for up to 72 h. Cells were visualized by staining for Rac and actin (phalloidin) as detailed in main text. Three different morphologies were identified: a) resting cells spread and adherent (“Resting”, yellow arrow); b) extended had a stellate fibroblast-like appearance (“Extended” green arrow); c) round showed limited substrate adherence as a prelude to complete detachment (“Round” blue arrow). (B) Quantification of three representative fields with 40–60 cells/field for control and R-naproxen treated samples are shown, with the same color convention used in A. Data show changes seen over time (24–48 h) post-EGF stimulation. 72 h samples were not quantified due to the large numbers of cells that had detached in the control sample by this time point. N = 3. **Figure G. R-Naproxen exhibits enantioselective inhibition of Rac1 in human ovarian cancer cells.** Human ovarian cancer (OvCa433) cells were serum starved (24 h in MEM containing 0.1% BSA) and left untreated or pretreated for 1 h with 300 μM R-Naproxen, S-Naproxen or 6-MNA prior to stimulation with 10 ng/ml EGF for 2 min. (Time course studies of Rac1 activation in OvCa433 cells measured by GLISA in response to EGF stimulation evidenced a 3-fold increase in activity above baseline with 2 min of treatment that was sustained 2-fold above baseline for up to 30 min of EGF treatment.) Rac1 activation by EGF in human ovarian cancer (OvCa433) cells was significantly inhibited (one-way ANOVA and Tukey’s multiple comparison test, ***p<0.001) by 300 μM R-naproxen in the presence of EGF as compared to the EGF stimulated control, and was not significantly different from unstimulated control (-EGF) with or without R-naproxen. In contrast, S-naproxen (ns, non-significant) and 6-MNA (*p<0.05) had no or limited effect on blocking Rac1 activation by EGF stimulation. Pairwise comparisons of baseline values –EGF, +/- drug treatment showed no significant differences. **Figure H. Virtual Screen for Drugs with alpha-methyl carboxylate identifies racemic ketorolac for further testing.** Differential activities in cell based studies of the chemically related structural series encompassing R-naproxen, S-naproxen and 6-MNA prompted focus on the α-methyl carboxylate as a critical structural determinant. With R-naproxen as query and focusing on α-methyl carboxylates, we evaluated all approved NSAIDs and several α-Me-COOH drugs (“rotational barrier” hypothesis). In total 39 NSAIDs and fifteen α-Me-COOH launched drugs were evaluated; ketorolac (separated enantiomers) was on the list, and the racemic is suggested as “compromise”. Heatmap shows the less-than-perfect overlap of the two queries, R-naproxen and R-ketorolac. **Table A. Summary Results of Primary Screen of Prestwick Library Tested at Single Dose against 8 GTPases.** The 2007 Prestwick Chemical Library was tested in multiplex format against Ras-related GTPases. All compounds were tested at a single dose against 888 compounds. Only four compounds, all NSAIDs, were found active. Twenty-three NSAIDs were tested in total as part of the library. **Table B. Summary of NSAID Primary and Confirmatory Screening Outcomes.** Dose response assays of all actives and select chemically related NSAIDs were conducted in multiplex format against eight GTPases. EC_50_ values are as indicated and only R-naproxen exhibited μM activity against Rac and Cdc42. **Table C. Enantiomer Selectivity of Cyclooxygenase Enzymes towards NSAIDs.** R-Naproxen, S-Naproxen, 6MNA, R-ketorolac, and S-ketorolac were comparatively evaluated for inhibitory activities against cyclooxygenase enzymes via in vitro assays. Table provides comparisons with published literature where available. **Table D. Results of the NSAID-focused Virtual Screen using R-naproxen as Query. Table E. Results of the LBVS Counter-screen.** Each of the queried compounds contains an alpha-methyl or a di-methyl carboxylate, respectively. The asterisk (*) indicates the presence of additional substituents to one, or both of the methyl groups linked to the carboxylate. Using low scores for both R-enantiomer queries, the LBVS counter-screen ruled out exatecan acid, S-flunoxaprofen, gemfibrozil, cinalukast, fexofenadine, and tanomastat. Compounds that remain of potential interest because their scores were above either R-naproxen or R-ketorolac and include cilomilast, R-flunoxaprofen, tiagabine, levocabastine, methallenestril, mitiglinide, cicloxilic acid, ecabet, S-ibuprofen, R-ibuprofen and S-naproxen. **Table F. Serum Concentrations and Effective Doses of Drugs and Drug-Like Molecules on GTPase Targets.** Serum concentrations (maximum (C_max_) and steady state (C_ave_)) were based on typical oral dosing (S-Naproxen 500 mg; R,S-ketorolac 30 mg; 6-MNA 750–20000 mg of nabumetone and derived from Roche Investigator’s brochure and primary literature. An IV dose of 30 mg ketorolac achieves a C_max_ of 13.7 μM. IC_50_ values for COX1/2 in human cells were obtained from the literature. IC_50_ of ketorolac isoforms determined via effector binding assay this manuscript. Migration IC_50_ values for NSAIDs were estimated from limited dose response data (this manuscript) or calculated by GraphPad Prism5 (ketorolac); data for CID2950007/ML141 are published. N/A-not applicable, no human dosing. ND = not detected. **Table G. NSAID Docking Scores and Analyses.** Docking studies were performed as detailed in main text and illustrated in Figs 9 and 10. The Generalized-Born Volume Integral/Weighted Surface area (GBVI/WSA dG) scoring function was implemented in the Molecular Operating Environment (MOE) software and used to estimate the free energy of binding of each ligand from a given pose, with lower scores corresponding to more favorable poses. Distances of Mg^2+^ from the carboxylate moiety of each docked compound are measured in Angstroms.
(PDF)
