# Supplementary material for: Novel Activities of Select NSAID R-Enantiomers against Rac1 and Cdc42 GTPases
Source: PLoS One. 2015 Nov 11;10(11):e0142182. doi: 10.1371/journal.pone.0142182 (PMC4641600; doi:10.1371/journal.pone.0142182)
Supplement: S1 File — Figure A. R-naproxen selectively inhibits BODIPY-GTP binding by Rac1 and Cdc42 in vivo. R-naproxen in contrast to S-naproxen and 6-methoxy naphthalene acid (6MNA) inhibited BODIPY-GTP binding to GSH bead immobilized GST-Rac1 and GST-Cdc42 GTPases in a dose-dependent manner. BODIPY-GTP concentration was held constant at 300 nM for these experiments and drug concentrations varied from 10 nM to 100 μM. Bead-associated fluorescence intensity was quantified by flow cytometry and used to monitor drug treatment induced changes in nucleotide binding. The inhibition curves were fitted to the sigmoidal dose-response equation using GraphPad Prism5. The EC50 of R-naproxen was 18 μM for both Rac1 and Cdc42, and an efficacy of 32% and 38%, respectively. The EC50 of S-naproxen was >200 μM for Rac1 and 4.2 μM for Cdc42 and the efficacy was 0% and 23%, respectively. 6MNA was inactive. N = 2. Figure B. NSAIDs without effect on guanine nucleotide binding. Dose response assays were conducted on eight Ras-related GTPases simultaneously using a multiplex format. Figure C. R-naproxen is not cytotoxic or anti-proliferative. (A-B) Ovarian cancer cells (OvCa433) were incubated for 48 h with R-naproxen or S-naproxen (0–300 μM) and lactate dehydrogenase (LDH) release into the media was determined by reduction of the tetrazolium salt INT into formazan relative to a positive control where membranes were permeabilized. (C-D) Effects on cell viability were measured by plating ovarian cancer cells (OvCa433) in 96-well plates in 10% FBS/MEM media and treating with R-naproxen or S-naproxen (0–300 μM) for 24–72 h. Viable cells were quantified using an MTS assay. (E) Proliferation of ovarian cancer cells (OvCa429 or OvCa433) treated with 0–300 μM each of R-naproxen, S-naproxen, 6 MNA or the Rac1 inhibitor, NSC23766 for a total of 6 days was measured using a BrdU assay. The results are from 3 independent experiments conducted in triplicate. Figure D. R-naproxen selectively causes the time dependent diss [file pone.0142182.s001.pdf]

Supplemental File S1

Supplemental Materials and Methods

Multiplex Screening Procedures and Dose Response Assays–Methods using 4 μm glutathione (GSH) conjugated beads for multiplex screening assays and dose response measurements are as given in main text.

In Vitro Nucleotide Binding Assays on Individual GTPases–Superdex GSH conjugated beads, 13 μm in diameter, were prepared and loaded with GST-GTPase as previously described [1]. At a maximal concentration of bound protein we estimate ~5x10^6 GST-GTPase molecules are bound to each bead, representing a concentration of ~2 nM bead-bound protein [2]. For individual dose-response measurements the nucleotide concentrations were fixed at the determined dissociation constants of Rac1 and Cdc42 for BODIPY-GTP ($K_d \approx$ 300 nM). Assays were performed in HEPES buffer (30 mM HEPES, pH 7.5, 20 mM NaCl, 100 mM KCl, 1 mM EDTA, 1 mM DTT, 0.01% (v/v) NP40, 0.1%BSA). For each measurement, GTPase loaded GSH beads (2 ×10^3 total beads) were pre-incubated with DMSO or increasing concentration of the compound for 15 min followed by incubation with BODIPY-GTP for 1 h at 4°C. For measurement, samples were diluted at least 10-fold and analyzed using a BD FACScan flow cytometer.

Cytotoxicity, Cell Viability and Proliferation Assays–For cytotoxicity tests, OvCa433 cells were plated in 96-well plates (8,000 cells/well) and cultured for 24 h in complete medium. Cells were treated with increasing doses (0-300μM) of R-naproxen or S-naproxen for 48 h in MEM containing 0.1% BSA. Cytotoxicity was measured using a LDH release assay (Cytotoxicity Detection Kit^{PLUS}, Roche Diagnostics GmbH, Germany) as compared to a detergent permeabilized positive control and formazan formation was measured at 490 nm using an ELISA plate reader.

For cell viability, OvCa433 cells were seeded into 96-well plates at a density of 30,000 cells/mL in 10% FBS/MEM media. Effects on cell viability were measured in 0.1% BSA/MEM after 24-48 h treatment with (0-300 μM) R-naproxen or S-naproxen in 96 well plates using an MTS (3-(4,5-dimethylthiazol-2-yl)-5-(3-carbo xymethoxyphenyl)-2-(4-sulfophenyl)-2H-tetrazolium) assay, which monitors metabolically active mitochondria.

Effects on ovarian cancer cell (OvCa429 and OvCa433) proliferation were measured using a BrdU assay (Millipore #2752, Billerica, MA). Cells were plated at 0.5 x 10^3 cells per well in 96-well plates (Corning #3596, Corning, NY) and allowed to adhere for 24h before treatment with varying concentrations of R-naproxen, S-naproxen, 6-methoxynaphthalenic Acid, and NSC23766. Media with or without treatments was replaced every two days. After a 5-day incubation period, cells were treated overnight with the BrdU reagent and fixed according the vendor's protocol. Each treatment was performed in triplicate and the assay was performed three times.

GEF Assays–Cdc42 and Rac1 guanine exchange factor (GEF) activity assays were carried out according to the protocol outlined in the RhoGEF exchange assay kit, BK100 (Cytoskeleton, Denver, CO, USA). Human Dbs GEF was from Cytoskeleton, Inc. HeLa cells were transfected with a myc-Tiam1 plasmid (kind gift of Dr. Channing Der, University of North Carolina, Chapel Hill) using LipofectamineTM 2000. Cell lysates were prepared and myc-Tiam1 was immunoprecipitated according to procedures stipulated in the c-myc tag IP/co-IP kit (Thermo Scientific, Pierce Biotechnology, Rockford USA). Phosphatidylinositol 4,5-bisphosphate (PIP2) is an important Tiam1 GEF activator based on its binding to the lipid homology domains of Tiam1 [3].

Phosphatidylinositol 4,5-bisphosphate (PIP2) lipid in $CHCl_3$: MeOH:H_2O mixture was dried under vacuum and then redispersed in immunoprecipitation elution buffer (50 μl) using intermittent bath sonication and vortexing. The equivalent of 10 μM PIP2 lipid (final) was incubated with 1 μM solution myc-Tiam1 (full length) for 5 min at room temperature as reported earlier [3] and then put on ice. His-tagged Rac1 (45 μM final) reconstituted in sterile water was mixed with nucleotide exchange buffer (20 mM Tris-HCl pH 7.5, 50 mM NaCl, 10 mM $MgCl_2$, 5% BSA and 0.75 μM mant-GTP) in a 100 μl volume (final) in a light sensitive 96 well plate. 1% DMSO (final), 100 μM R-naproxen in 1% DMSO (final) and 100 μM 6-MNA in 1% DMSO (final) were then added accordingly. The mixture was briefly shaken on a bench top shaker for 30 s before further incubation for 5 min at room temperature. Initial fluorescence measurements were recorded for 5 min on a 1420 Multichannel plate reader (PerkinElmer) by exciting mant-GTP at 360 nm and recording emitted fluorescence at 440 nm. PIP2 lipid bound Myc-Tiam1 (10 μM final) was then added to stimulate GEF-mediated Rac1 nucleotide exchanged and increases in fluorescence intensity monitored as a measure of mant-GTP binding. Control wells consisted of Rac1 in buffer only or with 1% DMSO.

GLISA Assays–Assay method as detailed in main text.

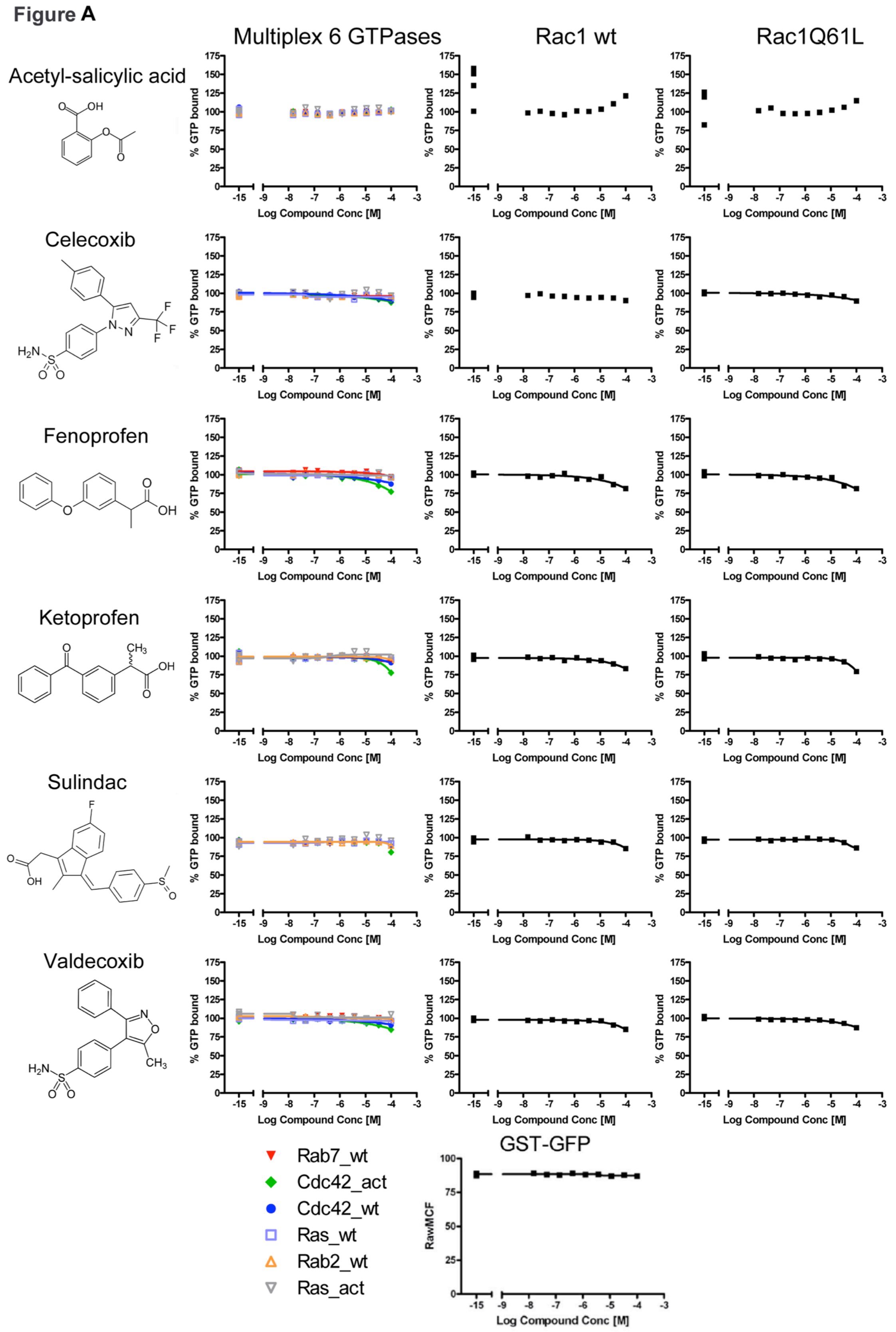

Figure A. NSAIDs without effect on guanine nucleotide binding. Dose response assays were conducted on six Ras-related GTPases simultaneously using a multiplex format. Rac1 wt and Rac1Q62L were measured in single-plex assays in buffer with EDTA and magnesium.

Figure B

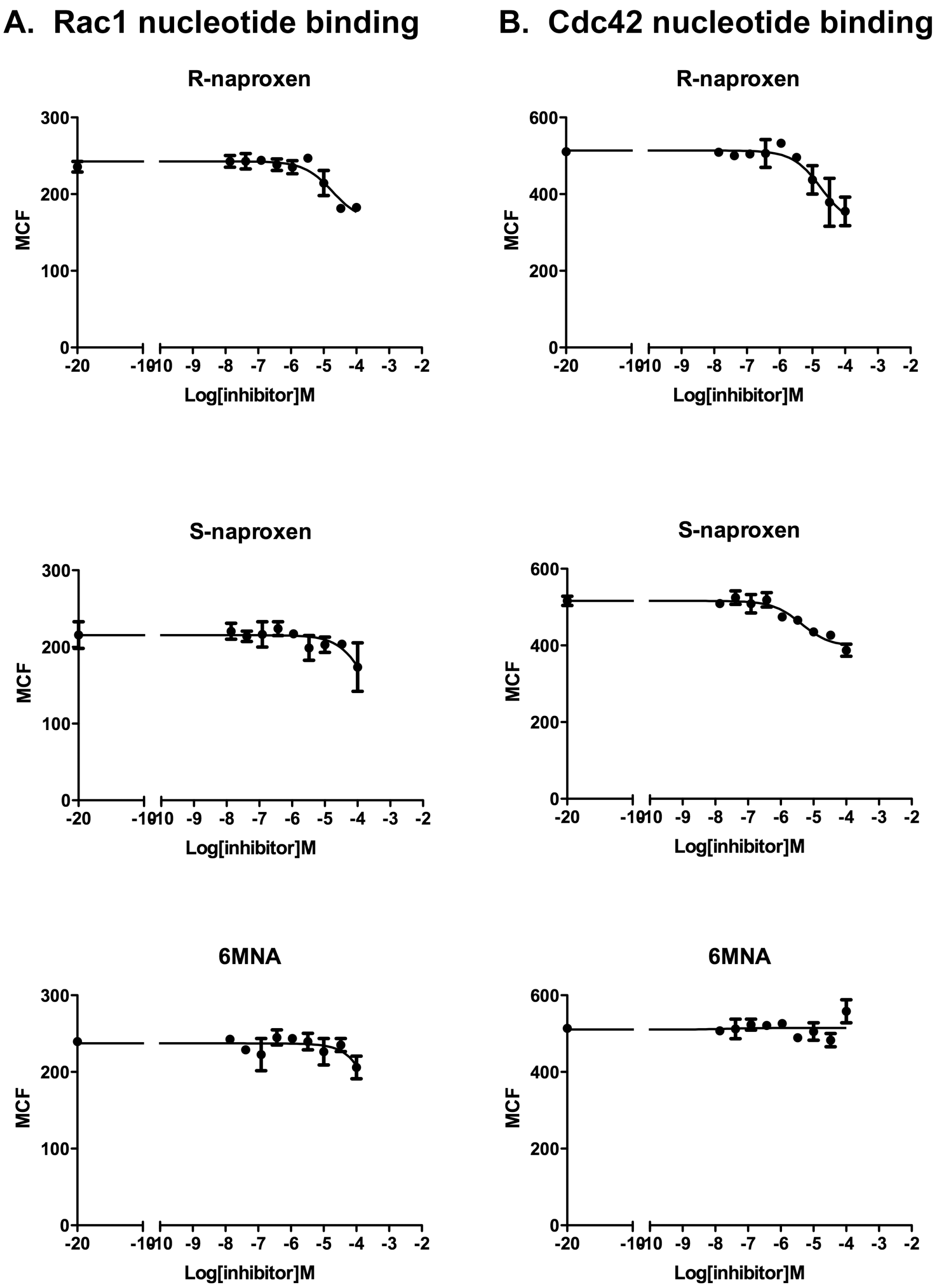

Figure B. R-naproxen selectively inhibits BODIPY-GTP binding by Rac1 and Cdc42 in vitro. R-naproxen in contrast to S-naproxen and 6-methoxy naphthalene acid (6MNA) inhibited BODIPY-GTP binding to GSH bead immobilized GST-Rac1 and GST-Cdc42 GTPases in a dose-dependent manner. BODIPY-GTP concentration was held constant at 300 nM for these experiments and drug concentrations varied from 10 nM to 100 μM. Bead-associated fluorescence intensity was quantified by flow cytometry and used to monitor drug treatment induced changes in nucleotide binding. The inhibition curves were fitted to the sigmoidal dose-response equation using GraphPad Prism5. The EC_{50} of R-naproxen was 18 μM for both Rac1 and Cdc42, and an efficacy of 32% and 38%, respectively. The EC_{50} of S-naproxen was >200 μM for Rac1 and 4.2 μM for Cdc42 and the efficacy was 0% and 23%, respectively. 6MNA was inactive. N=2.

Figure C

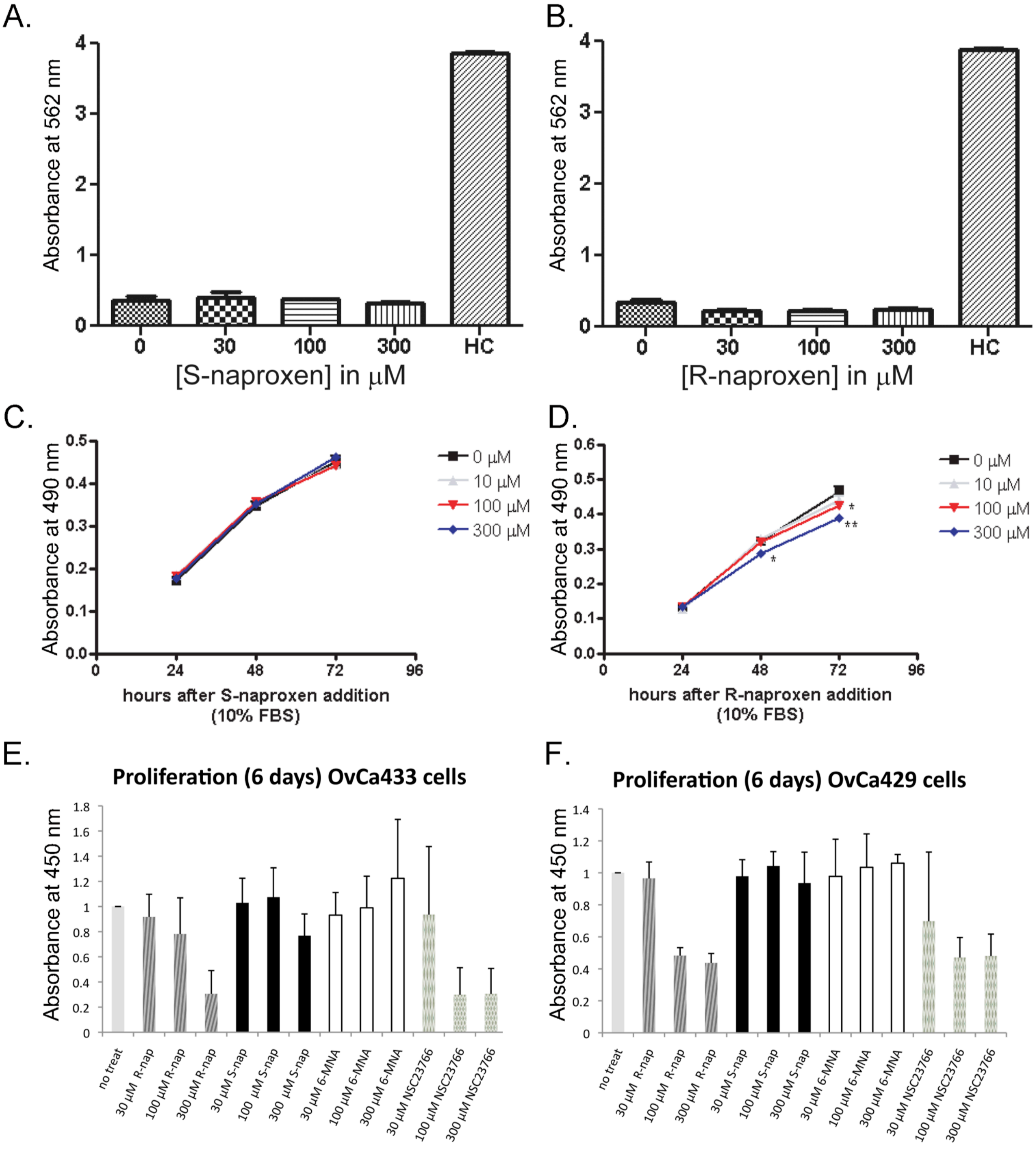

Figure C. R-naproxen is not cytotoxic or anti-proliferative. (A-B) Ovarian cancer cells (OvCa433) were incubated for 48 h with R-naproxen or S-naproxen (0-300 μM) and lactate dehydrogenase (LDH) release into the media was determined by reduction of the tetrazolium salt INT into formazan relative to a positive control where membranes were permeabilized. (C-D) Effects on cell viability were measured by plating ovarian cancer cells (OvCa433) in 96-well plates in 10% FBS/MEM media and treating with R-naproxen or S-naproxen (0-300 μM) for 24-72 h. Viable cells were quantified using an MTS assay. (E) Proliferation of ovarian cancer cells (OvCa429 or OvCa433) treated with 0-300 μM each of R-naproxen, S-naproxen, 6 MNA or the Rac1 inhibitor, NSC23766 for a total of 6 days was measured using a BrdU assay. The results are from 3 independent experiments conducted in triplicate. One-way ANOVA was conducted with Dunnett's multiple comparison post-test versus untreated controls. *$P<0.05$, **$P<0.01$

Figure D

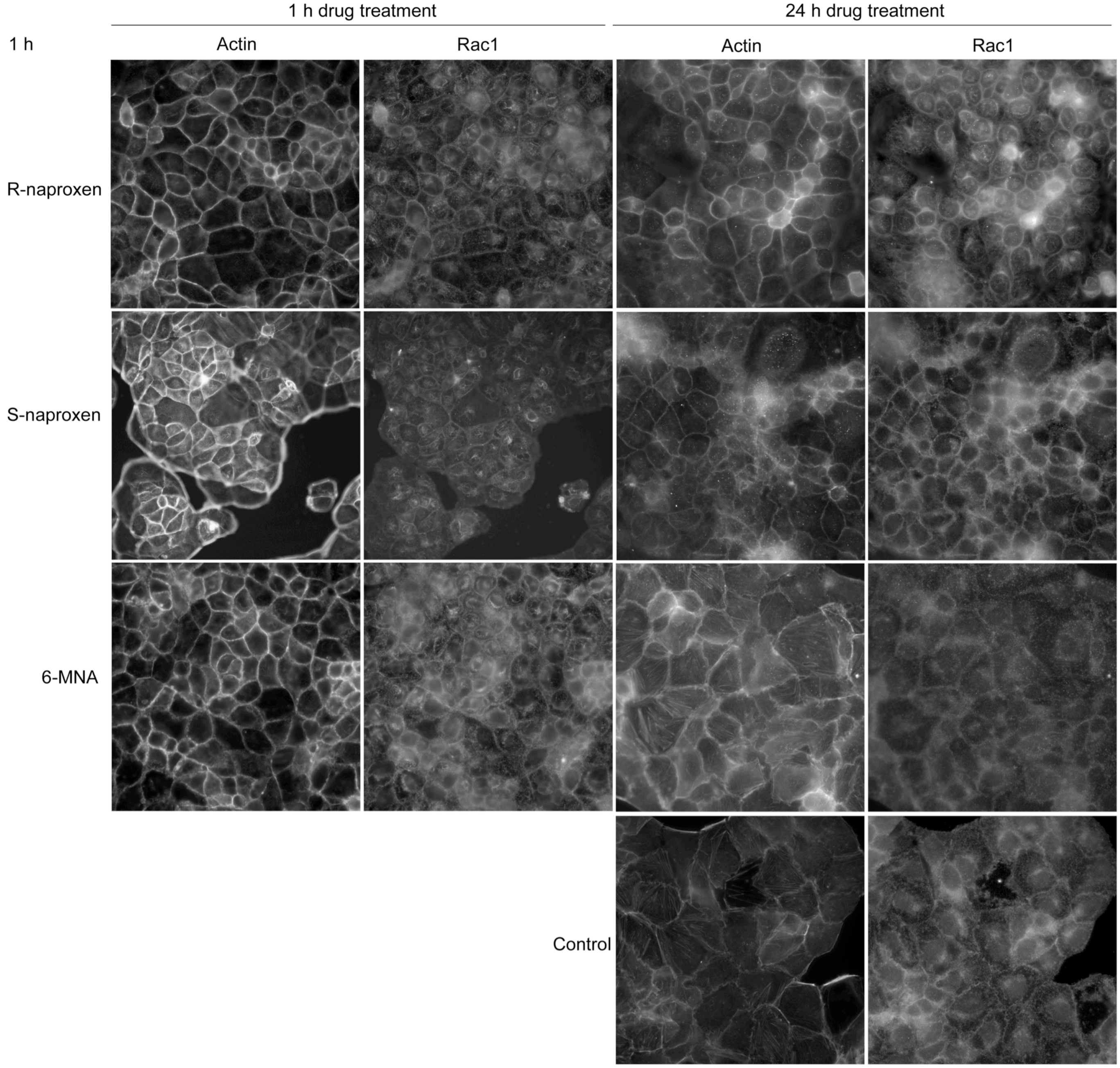

Figure D. R-naproxen selectively causes the time dependent dissociation of Rac1 from the plasma membrane. Ovarian cancer cells (OvCa433) were left untreated or treated with 300 μM R-naproxen, S-naproxen or 6MNA for 1 h or 24 h prior to paraformaldehyde fixation. Samples were labeled for actin using rhodamine phalloidin and immunostained for Rac1 (mAb from BD Transduction Labs 610650 see main text for details). Rac1 staining was lost from the cell borders and seen accumulated in the perinuclear region at 24 h in R-naproxen treated samples, but not in S-naproxen, 6 MNA or control samples. Images were acquired on a Zeiss Axioskop outfitted with a digital camera.

Figure E

A.

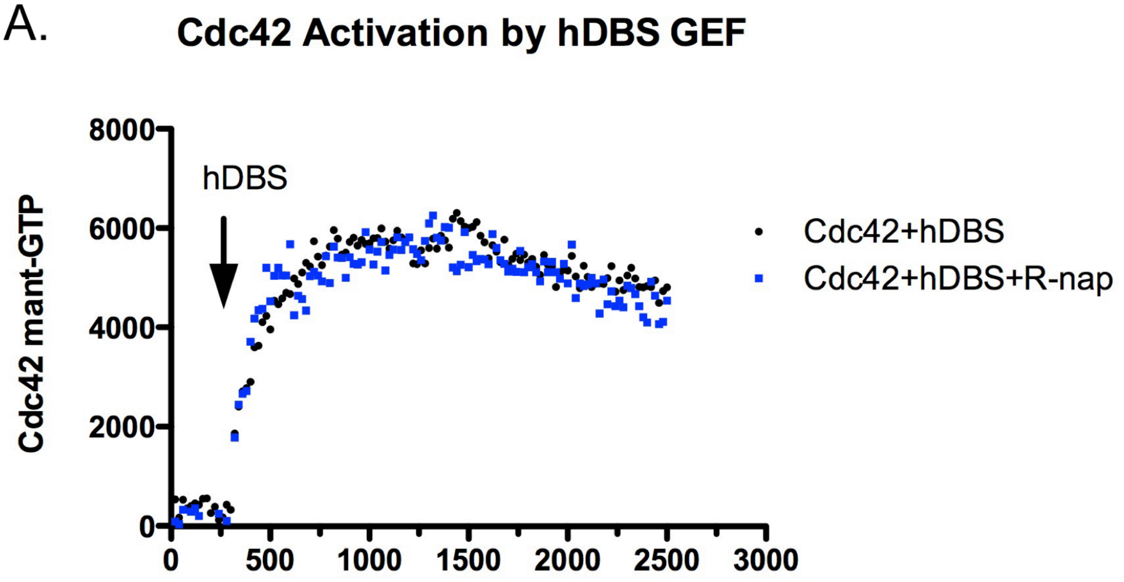

B.

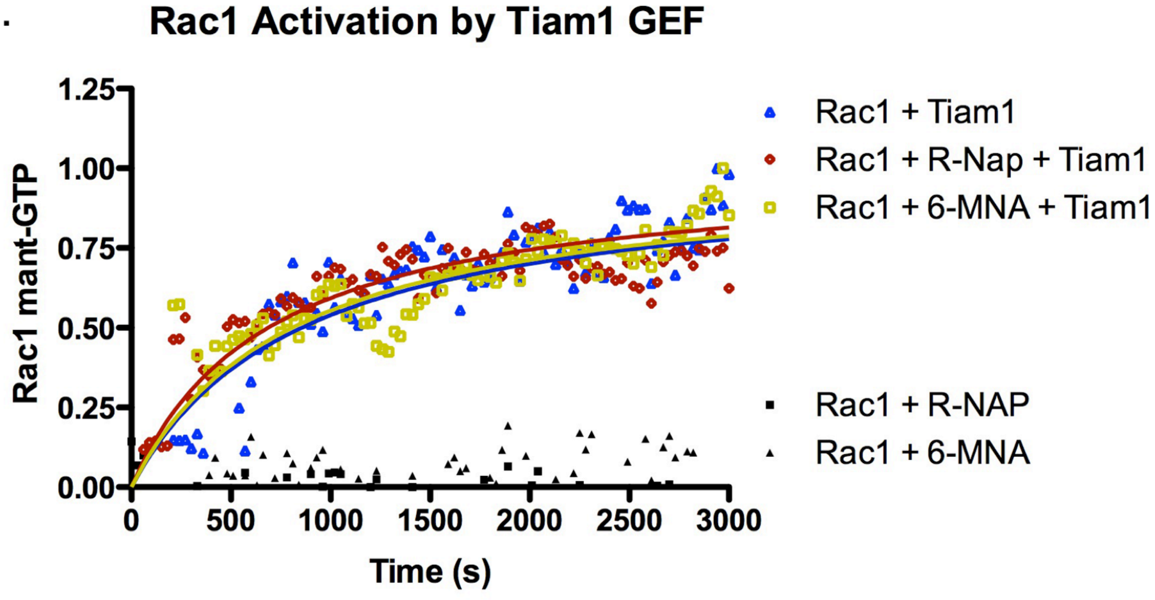

Figure E. R-naproxen does not inhibit in vitro GEF activity. (A) Real time measurement of mant-GTP binding in solution using purified Cdc42 and Dbs GEF domain with (blue squares) or without (black circles) 150 μM R-naproxen added. Plotted are data with control Cdc42 only values subtracted. Arrow denotes addition of hDBS GEF. (B) Real time measurement of mant-GTP binding in solution using purified Rac1 and PIP2/Tiam1 GEF. Plotted are fluorescence intensity measurements with DMSO or Rac1 only control values subtracted and then normalized to maximum fluorescence intensity. Curve fitting was performed using GraphPad Prism one site binding function. No differences in GEF-stimulated mant-GTP binding were observed in the presence of R-naproxen or 6-MNA relative to controls without drugs added.

Figure F

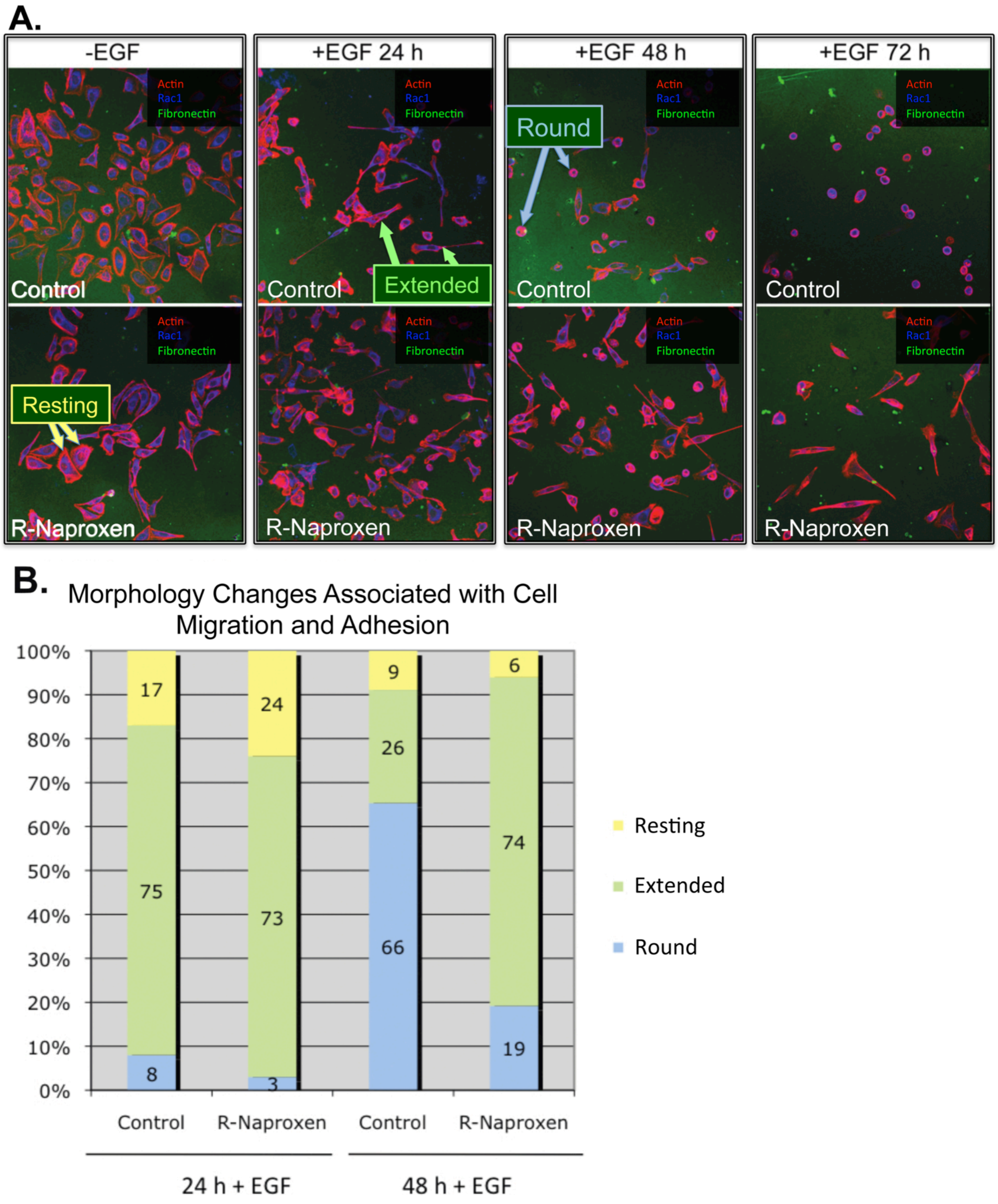

Figure F. R-naproxen reduces growth factor dependent cell morphologic changes necessary for cell migration and adhesion. (A) OvCa433 cells were plated on FITC- fibronectin and either left unstimulated or were stimulated for up to 72 h. Cells were visualized by staining for Rac and actin (phalloidin) as detailed in main text. Three different morphologies were identified: a) resting cells spread and adherent ("Resting", yellow arrow); b) extended had a stellate fibroblast-like appearance ("Extended" green arrow); c) round showed limited substrate adherence as a prelude to complete detachment ("Round" blue arrow). (B) Quantification of three representative fields with 40-60 cells/field for control and R-naproxen treated samples are shown, with the same color convention used in A. Data show changes seen over time (24-48 h) post-EGF stimulation. 72 h samples were not quantified due to the large numbers of cells that had detached in the control sample by this time point. N=3

Figure G

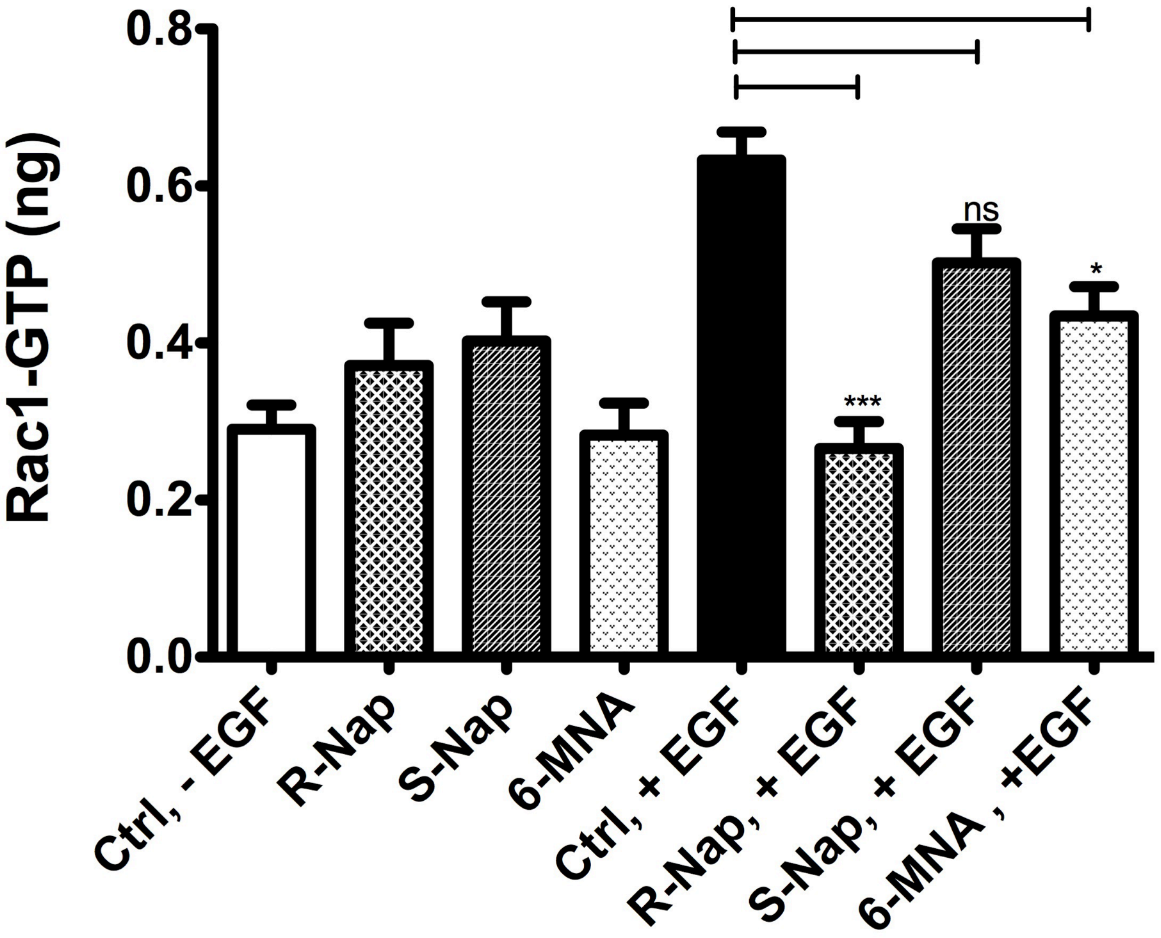

Figure G. R-Naproxen exhibits enantio-selective inhibition of Rac1 in human ovarian cancer cells. Human ovarian cancer (OvCa433) cells were serum starved (24 h in MEM containing 0.1% BSA) and left untreated or pretreated for 1 h with 300 μM R-Naproxen, S-Naproxen or 6-MNA prior to stimulation with 10 ng/ml EGF for 2 min. (Time course studies of Rac1 activation in OvCa433 cells measured by GLISA in response to EGF stimulation evidenced a 3-fold increase in activity above baseline with 2 min of treatment that was sustained 2-fold above baseline for up to 30 min of EGF treatment.) Rac1 activation by EGF in human ovarian cancer (OvCa433) cells was significantly inhibited (one-way ANOVA and Tukey's multiple comparison test, ***$p<0.001$) by 300 μM R-naproxen in the presence of EGF as compared to the EGF stimulated control, and was not significantly different from unstimulated control (-EGF) with or without R-naproxen. In contrast, S-naproxen (ns, non-significant) and 6-MNA (*$p<0.05$) had no or limited effect on blocking Rac1 activation by EGF stimulation. Pairwise comparisons of baseline values –EGF, +/- drug treatment showed no significant differences.

FigureH

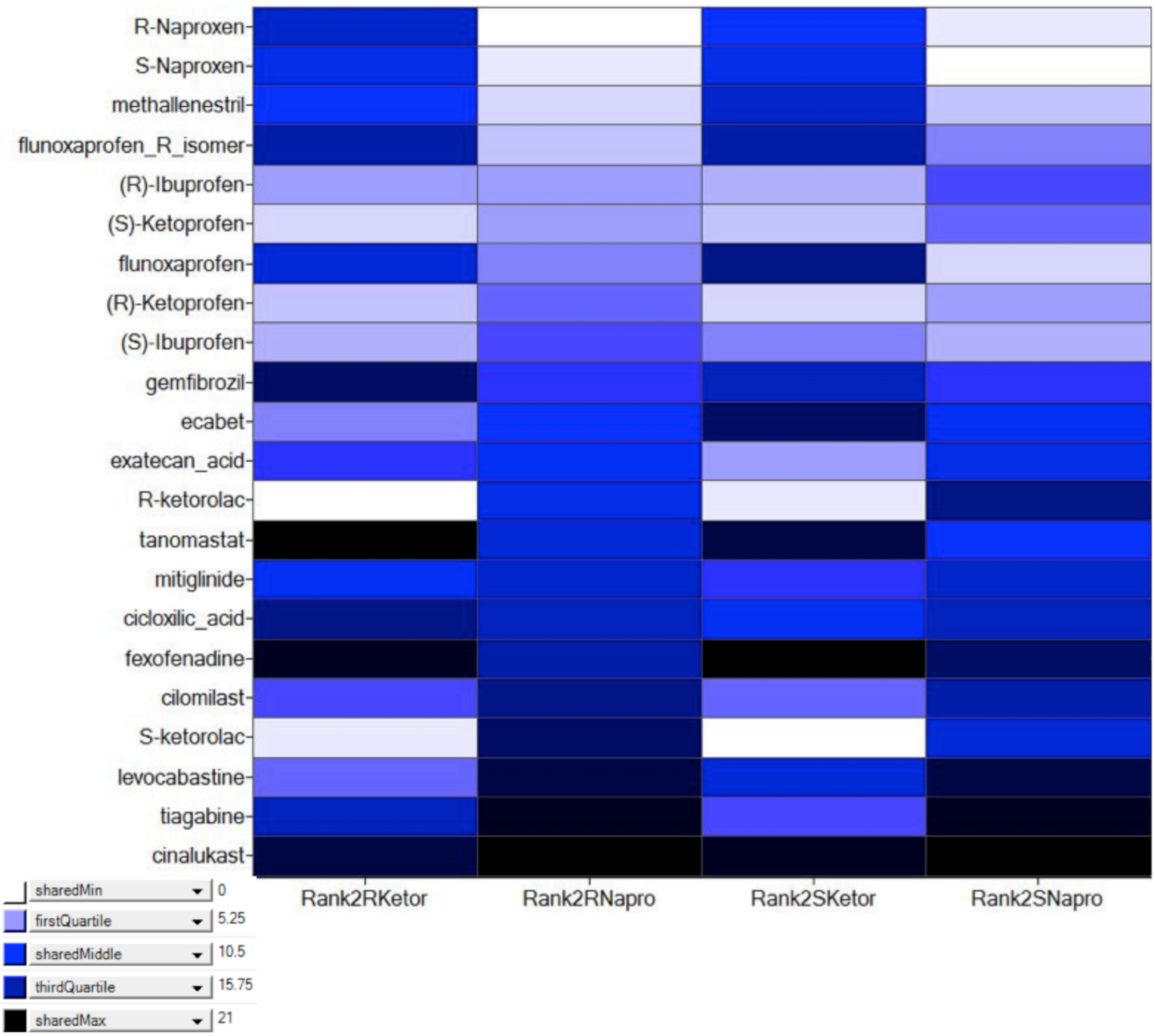

Figure H. Virtual Screen for Drugs with alpha-methyl carboxylate identifies racemic ketorolac for further testing. Differential activities in cell based studies of the chemically related structural series encompassing R-naproxen, S-naproxen and 6-MNA prompted focus on the α-methyl carboxylate as a critical structural determinant. With R-naproxen as query and focusing on α-methyl carboxylates, we evaluated all approved NSAIDs and several α-Me-COOH drugs ("rotational barrier" hypothesis). In total 39 NSAIDs and fifteen α-Me-COOH launched drugs were evaluated; ketorolac (separated enantiomers) was on the list, and the racemic is suggested as "compromise". Heatmap shows the less-than-perfect overlap of the two queries, R-naproxen and R-ketorolac.

Table A. Summary Results of Primary Screen of Prestwick Library Tested at Single Dose against 8 GTPases

Compound Identifying #	Structure	Compound Name (Prestwick Library 2007, 888 total compounds)	Primary Screen Hit*
**754**	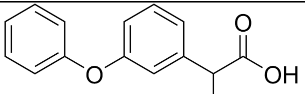	**Fenoprofen calcium salt dihydrate CC(C(O)=O)C1=CC(OC2=CC=CC=C2)=CC=C1**	**Active**
**219**	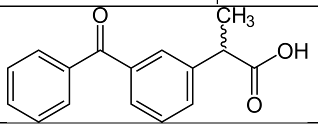	**Ketoprofen CC(C(O)=O)C1=CC(=CC=C1)C(=O)C1=CC=CC=C1**	**Active**
**45**	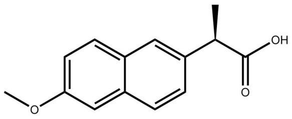	**R-Naproxen C[C@@H](c1ccc2cc(ccc2c1)OC)C(=O)O**	**Active**
**N/A**	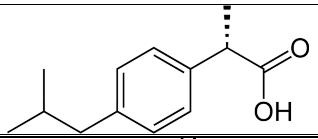	**S-(+)-Ibuprofen**<br>CC(C)CC1=CC=C(C=C1)[C@H](C)C(O)=O	**Active**
868	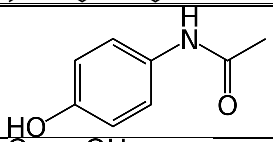	Acetaminophen CC(=O)NC1=CC=C(O)C=C1	Inactive
98	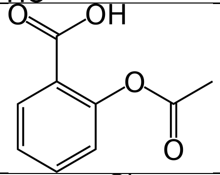	Acetylsalicylsalicylic acid<br>CC(=O)OC1=C(C=CC=C1)C(=O)OC1=C(C=CC=C1)C(O)=O	Inactive
594	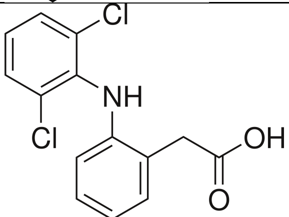	Diclofenac sodium OC(=O)CC1=C(NC2=C(Cl)C=CC=C2Cl)C=CC=C1	Inactive
39	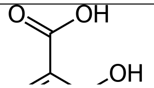	Diflunisal OC(=O)C1=C(O)C=CC(=C1)C1=C(F)C=C(F)C=C1	Inactive

231	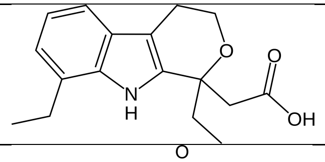	Etodolac CCC1=CC=CC2=C1NC1=C2CCOC1(CC)CC(O)=O	Inactive
218	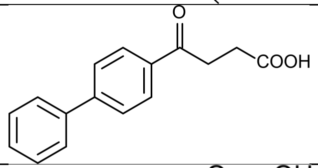	Fenbufen OC(=O)CCC(=O)C1=CC=C(C=C1)C1=CC=CC=C1	Inactive
203	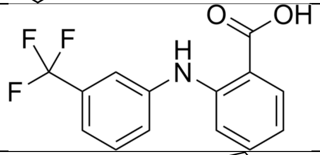	Flufenamic acid OC(=O)C1=C(NC2=CC(=CC=C2)C(F)(F)F)C=CC=C1	Inactive
272	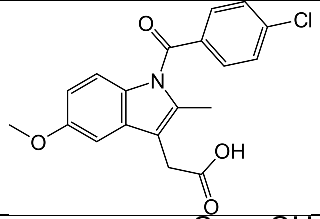	Indomethacin COC1=CC2=C(C=C1)N(C(=O)C1=CC=C(Cl)C=C1)C(C)=C2CC(O)=O	Inactive
206	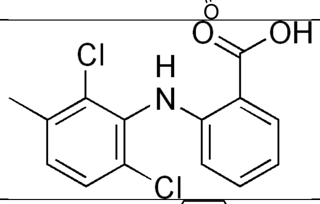	Meclofenamic acid sodium salt monohydrate CC1=C(Cl)C(NC2=C(C=CC=C2)C(O)=O)=C(Cl)C=C1	Inactive
54	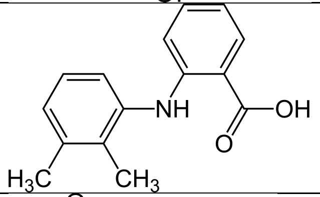	Mefenamic acid CC1=CC=CC(NC2=C(C=CC=C2)C(O)=O)=C1C	Inactive
194	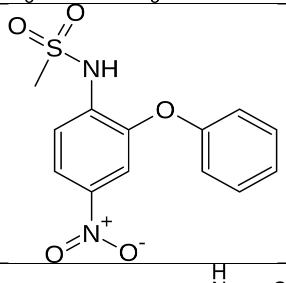	Nimesulide CS(=O)(=O)NC1=C(OC2=CC=CC=C2)C=C(C=C1)[N+]([O-])=O	Inactive
533	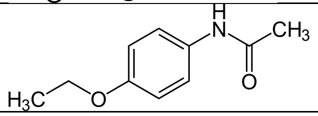	Phenacetin CCOC1=CC=C(NC(C)=O)C=C1	Inactive

211	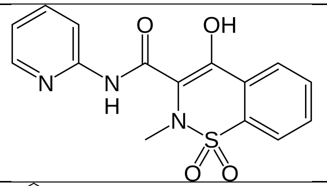	Piroxicam CN1C(C(=O)NC2=NC=CC=C2)=C(O)C2=C(C=CC=C2)S1(=O)=O	Inactive
520	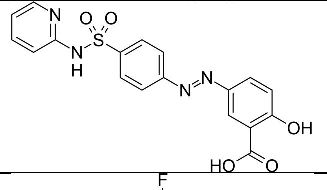	Sulfasalazine OC(=O)C1=CC(=CC=C1O)\N=N\C1=CC=C(C=C1)S(=O)(=O)NC1=CC=CC=N1	
73	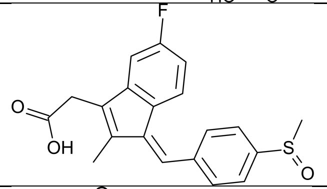	Sulindac	Inactive
816	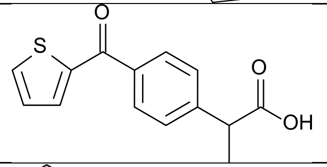	Suprofen CC(C(O)=O)C1=CC=C(C=C1)C(=O)C1=CC=CS1	Inactive
527	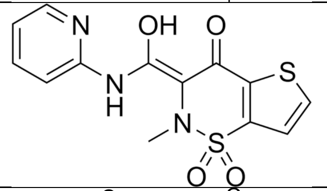	Tenoxicam CN1C(C(=O)NC2=NC=CC=C2)=C(O)C2=C(C=CS2)S1(=O)=O	Inactive
496	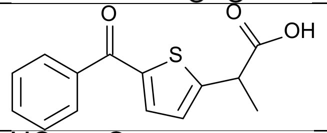	Tiaprofenic acid CC(C(O)=O)C1=CC=C(S1)C(=O)C1=CC=CC=C1	Inactive
205	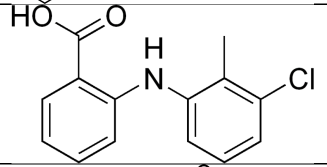	Tolfenamic acid CC1=C(Cl)C=CC=C1NC1=C(C=CC=C1)C(O)=O	Inactive
856	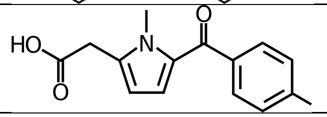	Tolmetin sodium salt dihydrate CN1C(CC(O)=O)=CC=C1C(=O)C1=CC=C(C)C=C1	Inactive

*Active: >20% change in BODIPY-GTP binding on any one GTPase at 10 μM concentration. Inactive: <20% change in BODIPY-GTP binding on any one GTPase at 10 μM concentration.

Table A Legend. The 2007 Prestwick Chemical Library was tested in multiplex format against Ras-related GTPases. All compounds were tested at a single dose against 888 compounds. Only four compounds, all NSAIDs, were found active. Twenty-three NSAIDs were tested in total as part of the library.

Table B. Summary of NSAID Primary and Confirmatory Screening Outcomes.

Compound Name/SMILES	Rac1 wt (act) EC50, M[b]	Cdc42 wt (act) EC50, M[b]	H-Ras wt (act) EC50, M[b]	Rab2 EC50, M[b]	Rab7 EC50, M[b]	GLISA Rac1[c]	GLISA Cdc42[c]	Rac protein expression[d]	Rac PM Localiz.[e]	Tiam1 GEF PM localiz.[e]
**ACTIVES**										
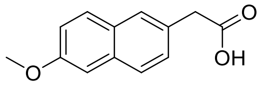 6-methoxy naphthalene acetic acid	inactive (inactive)	inactive (1.23E-05)	inactive (inactive)	inactive	inactive	0% inhibition at 300 µM	0% inhibition at 300 µM	No change	98%	73%
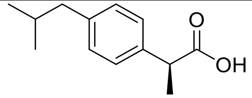 **S-(+)-Ibuprofen***	inactive (inactive)	inactive (6.41E-06)	inactive (inactive)	inactive	inactive				78%**	
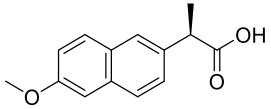 **R-Naproxen***	2.50E-06 (2.32E-06)	2.82E-06 (2.61E-06)	inactive (inactive)	inactive	inactive	100% inhibition at 300 µM	100% inhibition at 150 µM	No change	34%	32%
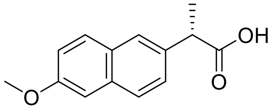 S-Naproxen	1.17E-05 (inactive)	1.05E-05 (inactive)	inactive (inactive)	inactive	inactive			No change	87%	65%
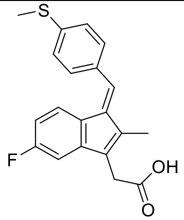 Sulindac sulfide	5.4E-05 (5.3E-05)	2.0E-04 (4.86E-05)	inactive (inactive)	inactive	inactive					
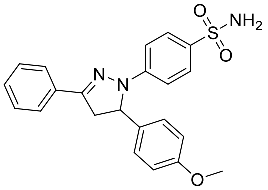 CID2950007/ML141	inactive (inactive)	2.6E-06 (5.4E-06)	inactive (inactive)	inactive	inactive					
**INACTIVES**										

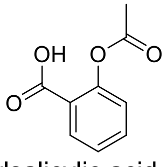 Acetylsalicylic acid (Aspirin)	inactive (inactive)	inactive (inactive)	inactive (inactive)	inactive	inactive					
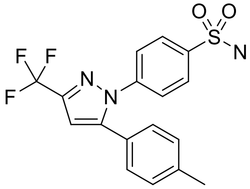 Celecoxib (celebrex)	inactive (inactive)	inactive (inactive)	inactive (inactive)	inactive	inactive					
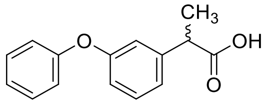 **Fenoprofen***	Inactive (inactive)	inactive (inactive)	inactive (inactive)	inactive	inactive					
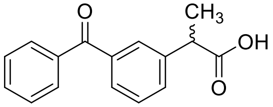 **Ketoprofen***	inactive (inactive)	inactive (inactive)	inactive	inactive	inactive				45%**	
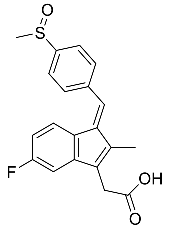 Sulindac	inactive (inactive)	inactive (inactive)	inactive (inactive)	inactive	inactive					
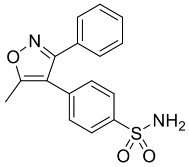 Valdecoxib (bextra)	inactive (inactive)	inactive (inactive)	inactive (inactive)	inactive	inactive					

Table B Legend. Dose response assays of all actives and select chemically related NSAIDs were conducted in multiplex format against eight GTPases. EC_{50} values are as indicated and only R-naproxen exhibited μM activity against Rac and Cdc42.

[a]Primary screen was performed on 2007 Prestwick Chemical Library® containing 888 compounds of which 18 are classified as NSAIDs (see Table S1 for details). Compounds are categorized as active if they changed BODIPY-GTP-binding (activators-increase binding; inhibitors-decrease binding) on one of eight GTPases in primary or secondary multiplex assay by greater than 20% at concentrations less than or equal to $1x10^{-4}$ M; GTPases tested were Cdc42 wild-type (wt), Cdc42 activated mutant (act), Rab2, Rab7, Rac wt, Rac act, Ras wt, Ras act. *NSAIDs identified in the primary screen.

[b]Confirmatory screens entailed dose response assays conducted in a multiplex format with eight GTPases tested as for primary screen. BODIPY-GTP-binding activity was measured in the presence of 10 nM to 100 μM of each of 11 test compounds (4 primary screen hits and 7 additional NSAIDs considered to have chemical similarity to GTPase active compounds). GST-GFP coated beads served as a control to detect non-specific dissociation or denaturation caused by compound addition–none of the tested NSAIDs had any effect on bead-GST-protein binding. EC_{50} values were determined using Prism to determine dose response fits as shown in Figure 1 of main text; fits have R2 > 0.9. Active compounds based on same definition used as in primary screen[a].
[c]GLISA measures the cellular activation status of Rac1 and Cdc42 based on Pak effector protein binding.
[d]Protein expression levels were measured by immunoblot analysis.
[e]Changes in localization were monitored by immunofluorescence and quantified using ImageJ, **assays performed with 0.1% DMSO
ND not determined.
‡A well-characterized selective Cdc42 inhibitor served as a positive control.

Table C. Enantiomer Selectivity of Cyclooxygenase Enzymes towards NSAIDS

Compound	COX-1<br>IC_{50} (μM)	COX-2<br>IC_{50} (μM)	COX-1<br>IC_{50} (μM)	COX-2<br>IC_{50} (μM)
	CEREP		Previously Published	
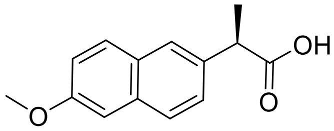<br>R-Naproxen*	7.20	4.20	R>S[3]<br>>25[4]	>25[4]
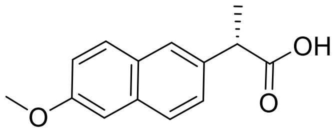<br>S-Naproxen	.059	.07	>25[4]<br>0.34[5]<br>0.6[6]<br>4.8[7]<br>72.4[8]<br>48.3[9]	0.9[4]<br>0.18[5]<br>2.0[6]<br>28.4[7]<br>30.7[8]<br>79.5[9]
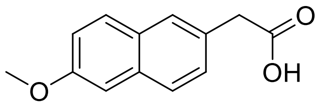<br>6-methoxy naphthalene acetic acid	28.0	>100[1]	64[7]<br>149[10]<br>278[11]<br>2.29[12]<br>1.3[13]<br>42[14]	93.5[7]<br>230[10]<br>187[11]<br>~5[12]<br>146[14]
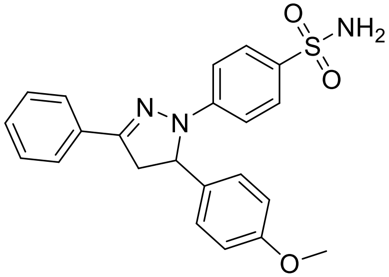<br>CID2950007/ML141	1.80	0.58	NA	NA
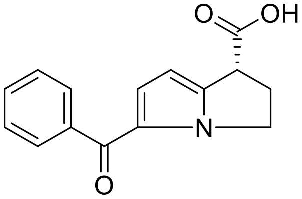<br>R-Ketorolac	0.99	>100[2]	>100[15]<br>>100[16]<br>Not sig[17]<br>36.4[18]	>100[15]<br>>100[16]<br>Not sig[17]<br>Not sig[18]
	<0.01	0.085	0.1[15]	0.79[15]

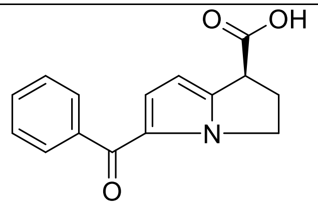<br>S-Ketorolac			0.46[16]<br>0.1[17]<br>0.017[18]	1.46[16]<br>2.7[17]<br>3.3[18]

Table C Legend. R-Naproxen, S-Naproxen, 6MNA, R-ketorolac, and S-ketorolac were comparatively evaluated for inhibitory activities against cyclooxygenase enzymes via in vitro assays. Table provides comparisons with published literature where available.

[1]> concentration: IC_{50} value above the highest test concentration. Concentration-response curve shows less 50% effect at the highest tested concentration.
[2]< concentration: IC_{50} value below the lowest test concentration. Concentration-response curve shows more 50% effect at the lowest tested concentration.
[3]IC_{50} values were not calculated. COX-1 inhibition was estimated by production of thromboxane B2 from collagen-stimulated human platelets [4].
[4]IC_{50} values were obtained for purified ovine COX-1 and expressed, recombinant murine COX-2 using saturating concentration of substrate [5].
[5]IC_{50} values were obtained for purified ovine COX-1 and expressed, recombinant murine COX-2 using sub-saturating concentration of substrate [5].
[6]IC_{50} values were measured using recombinant COX-1 and COX-2 produced in the bacculovirus system [6].
[7]IC_{50} values were measured using microsomal membranes prepared from sham-transfected *cos-1* cells or cells expressing human COX-1 or COX-2 [7].
[8]IC_{50} values were obtained for the inhibition of prostaglandin E2 (PGE2) and thromboxane B2 (TXB2) by naproxen in vitro using rat blood [8].
[9]IC_{50} values were obtained for the inhibition of prostaglandin E2 (PGE2) and thromboxane B2 (TXB2) by naproxen in vitro using human blood [8].
[10]IC_{50} values were measured in human peripheral monocytes stimulated with or without lipopolysaccharide for determine COX-2 and COX-1 activity, respectively [9].
[11]IC_{50} values were measured in cells isolated from human whole blood. Platelets were used for COX-1 and LPS-induced monocytes were used for COX-2 [10].
[12]IC_{50} values measured using enzyme purified from CHO cells stably transfected with human COX-1 and COX-2. COX-2 titrations did not achieve complete inhibition [11].
[13]IC_{50} value was obtained using U937 microsomes [11].
[14]IC_{50} values were obtained from human whole blood followed by measurement of COX-1 and COX-2 activity [12].

[15]IC_{50} values were measured using rat COX-1 and COX-2 expressed in a baculovirus system [13].

[16]IC_{50} values were measured using human COX-1 and COX-2 expressed in a baculovirus system [13].

[17]IC_{50} values were measured using purified ovine COX-1 and COX-2 from seminal vesicles and placenta [14].

[18]IC_{50} values for COX activity were measured from enzyme purified from ovine seminal vesicles or calcium ionophore stimulated human whole blood [15].

Table D. Results of the NSAID-focused Virtual Screen using R-naproxen as Query.

Drug Name	PCL[1]	2D Tan	Shape Tan	Scaled Color	LBVS Score
6-MNA	N	0.85	0.97	0.85	0.91
nabumetone	N	0.67	0.91	0.53	0.78
fenoprofen	Y	0.76	0.80	0.71	0.77
suprofen	Y	0.68	0.82	0.71	0.75
flurbiprofen	N	0.6	0.86	0.71	0.74
ibuprofen	N	0.66	0.79	0.71	0.73
alclofenac	N	0.59	0.84	0.57	0.71
tiaprofenic acid	Y	0.62	0.71	0.71	0.67
pirprofen	N	0.44	0.82	0.71	0.65
ketoprofen	Y	0.68	0.60	0.71	0.64
indobufen	N	0.45	0.76	0.59	0.62
fenbufen	Y	0.5	0.70	0.53	0.60
phenacetin	Y	0.43	0.78	0.28	0.59
tolfenamic acid	Y	0.42	0.72	0.52	0.58
tolmetin	Y	0.39	0.72	0.56	0.57
indomethacin	Y	0.42	0.68	0.56	0.56
mefenamic acid	Y	0.43	0.67	0.56	0.56
acetaminophen	Y	0.38	0.76	0.29	0.56
meclofenamic acid	Y	0.41	0.64	0.57	0.54
diclofenac	N	0.38	0.65	0.51	0.53
ketorolac	N	0.37	0.66	0.48	0.53
tenoxicam	Y	0.27	0.78	0.27	0.53
flufenamic acid	Y	0.4	0.61	0.56	0.52
oxaprozin	N	0.35	0.64	0.58	0.52
rofecoxib	N	0.38	0.63	0.51	0.52
phenazopyridine	Y	0.12	0.87	0.28	0.51
piroxicam	Y	0.28	0.75	0.26	0.51
flutamide	Y	0.28	0.65	0.38	0.48
sulindac	Y	0.47	0.45	0.57	0.47
valdecoxib	N	0.26	0.62	0.53	0.47
nepafenac	N	0.26	0.65	0.39	0.47
meloxicam	N	0.27	0.61	0.46	0.46
nimesulide	Y	0.3	0.57	0.49	0.45
etoricoxib	N	0.27	0.58	0.50	0.45
oxyphenbutazone	N	0.27	0.58	0.43	0.44
proquazone	N	0.33	0.52	0.43	0.43
celecoxib	N	0.22	0.60	0.42	0.43
phenylbutazone	N	0.25	0.56	0.29	0.41

[1]The Prestwick Chemical Library (PCL) column indicates presence or absence in the 2007 collection.

Table E. Results of the LBVS Counter-screen [16].

Name	Type	Priority	R-ketorolac	S-ketorolac	R-naproxen	S-naproxen
R-ketorolac	α-Me	high	1.000	0.857	0.540	0.547
S-ketorolac	α-Me	high	0.856	1.000	0.547	0.540
S-ketoprofen	α-Me	high	0.679	0.695	0.711	0.642
R-ketoprofen	α-Me	high	0.639	0.671	0.641	0.718
cilomilast	di-Me(*)	medium	0.583	0.564	0.515	0.510
R-flunoxaprofen	α-Me	medium	0.572	0.563	0.705	0.667
tiagabine	di-Me(*)	medium	0.552	0.558	0.440	0.446
levocabastine	di-Me(*)	medium	0.548	0.511	0.395	0.371
methallenestril	di-Me	medium	0.546	0.526	0.763	0.693
mitiglinide	di-Me(*)	medium	0.541	0.537	0.446	0.487
cicloxilic_acid	di-Me(*)	medium	0.536	0.508	0.582	0.576
ecabet	di-Me(*)	medium	0.529	0.512	0.545	0.518
S-ibuprofen	α-Me	medium	0.527	0.508	0.722	0.771
R-naproxen	α-Me	high	0.516	0.508	1.000	0.939
R-ibuprofen	α-Me	medium	0.510	0.528	0.771	0.718
S-naproxen	α-Me	medium	0.508	0.517	0.939	1.000
exatecan acid	di-Me(*)	low	0.507	0.518	0.473	0.479
S-flunoxaprofen	α-Me	low	0.498	0.577	0.668	0.705
gemfibrozil	di-Me	low	0.480	0.520	0.643	0.657
cinalukast	di-Me(*)	low	0.470	0.448	0.377	0.401
fexofenadine	di-Me	low	0.460	0.452	0.376	0.393
tanomastat	di-Me(*)	low	0.423	0.427	0.509	0.549

Table E Legend. Each of the queried compounds contains an alpha-methyl or a di-methyl carboxylate, respectively. The asterisk (*) indicates the presence of additional substituents to one, or both of the methyl groups linked to the carboxylate. Using low scores for both R-enantiomer queries, the LBVS counter-screen ruled out exatecan acid, S-flunoxaprofen, gemfibrozil, cinalukast, fexofenadine, and tanomastat. Compounds that remain of potential interest because their scores were above either R-naproxen or R-ketorolac and include cilomilast, R-flunoxaprofen, tiagabine, levocabastine, methallenestril, mitiglinide, cicloxilic acid, ecabet, S-ibuprofen, R-ibuprofen and S-naproxen.

Table F. Serum Concentrations and Effective Doses of Drugs and Drug-Like Molecules on GTPase Targets.

**Drug**	**R-Naproxen**	**S-Naproxen**	**6-MNA**	**(R,S)-Ketorolac**	**R-Ketorolac**	**S-Ketorolac**	**NSC23766**	**CID2950007/ML141**
**Target**	Rac1/Cdc42>COX	COX 1/2 >	COX 1/2	COX1>COX2	Rac1/Cdc42	COX1>COX2	Rac1	Cdc42
**Serum C_{max}**	N/A	97.4 μg/ml (413 ± 74 μM)[3]	41.2 μg/ml (58 μM)	6.4 ± 1.8 μM	N/A	N/A	N/A	N/A
**Serum C_{ave}**	N/A	130-391 μM	172-231 μM	8.3 ± 2.3 μM	N/A	N/A	N/A	N/A
**IC_{50} Rac1**	2.50 μM	>10 μM	inactive	----------	0.574	>100	~50 μM	ND
**IC_{50} Cdc42**	2.82 μM	>10 μM	inactive	----------	1.07	>100	ND	2.1-2.6 μM
**IC_{50} migration Rac1/Cdc42 dependent (est)**	~100 μM	≥300 μM	>300 μM	34 μM	7 μM	>100 μM	<30 μM	~10 μM

Table F Legend. Serum concentrations (maximum (C_{max}) and steady state (C_{ave})) were based on typical oral dosing (S-Naproxen 500 mg; R,S-ketorolac 30 mg; 6-MNA 750-20000 mg of nabumetone and derived from Roche Investigator's brochure and primary literature (S-Naproxen, [17-19]; ketorolac [13, 14, 20-22]; 6-MNA [9, 10, 21, 23]. An IV dose of 30 mg ketorolac achieves a C_{max} of 13.7 μM [22, 24]. IC_{50} values for COX1/2 in human cells were obtained from the literature (R and S-naproxen, [4, 8, 25, 26]; R and S-Ketorolac, [13-15, 20, 22, 24]***;*** 6-MNA, [9, 10, 21], NSC23766, [27]. IC_{50} of ketorolac isoforms determined via effector binding assay this manuscript. Migration IC_{50} values for NSAIDs were estimated from limited dose response data (this manuscript) or calculated by GraphPad Prism5 (ketorolac); data for CID2950007/ML141 are from [28]. N/A-not applicable, no human dosing. ND=not detected.

Table G NSAID Docking Scores and Analyses

	Rac1		Cdc42	
	Free Energy of Ligand Binding (GBVI/WSA dG) Kcal/Mol	NSAID carboxylate-Mg2+ dist (Å)	Free Energy of Ligand Binding (GBVI/WSA dG) Kcal/Mol	NSAID carboxylate-Mg2+ dist (Å)
6-MNA	-2.24	2.28	-1.33	2.38
R-ketorolac	-2.53	2.17	-2.19	2.18
S-ketorolac	-2.40	2.34	-1.98	2.70
R-naproxen	-2.54	2.25	-1.46	2.34
S-naproxen	-2.27	2.26	-1.33	2.37
6-MNA amide	-2.07	2.31	-2.47	2.46
R-ketorolac amide	-2.81	2.36	-2.48	2.39
S-ketorolac amide	-2.34	2.44	-2.46	2.34
R-naproxen amide	-2.65	2.38	-2.47	2.46
S-naproxen amide	-2.31	2.34	-2.33	2.40

Table G Legend. Docking studies were performed as detailed in main text and illustrated in Fig 9-10. The Generalized-Born Volume Integral/Weighted Surface area (GBVI/WSA dG) scoring function was implemented in the Molecular Operating Environment (MOE) software and used to estimate the free energy of binding of each ligand from a given pose, with lower scores corresponding to more favorable poses. [29, 30]. Amide derivatives were used as a control. Distances of Mg^{2+} from the carboxylate moiety of each docked compound are measured in Angstroms. Interaction distances were shortest for the R-carboxylate derivatives relative to the corresponding S-enantiomers and amide derivatives, affirming a preference for the R-carboxyl derivatives.

Supplemental References

1. Agola JO, Sivalingam D, Cimino DF, Simons PC, Buranda T, Sklar LA, et al. Quantitative bead-based flow cytometry for assaying Rab7 GTPase interaction with the Rab-interacting lysosomal protein (RILP) effector protein. Methods Mol Biol. 2015;1298:331-54. doi: 10.1007/978-1-4939-2569-8_28. PubMed PMID: 25800855.
2. Schwartz SL, Tessema M, Buranda T, Pylypenko O, Rak A, Simons PC, et al. Flow cytometry for real-time measurement of guanine nucleotide binding and exchange by Ras-like GTPases. Anal Biochem. 2008;381(2):258-66. doi: 10.1016/j.ab.2008.06.039. PubMed PMID: 18638444; PubMed Central PMCID: PMC2633595.
3. Crompton AM, Foley LH, Wood A, Roscoe W, Stokoe D, McCormick F, et al. Regulation of Tiam1 nucleotide exchange activity by pleckstrin domain binding ligands. J Biol Chem. 2000;275(33):25751-9. doi: 10.1074/jbc.M002050200. PubMed PMID: 10835422.
4. Kean WF, Lock CJ, Rischke J, Butt R, Buchanan WW, Howard-Lock H. Effect of R and S enantiomers of naproxen on aggregation and thromboxane production in human platelets. J Pharm Sci. 1989;78(4):324-7. PubMed PMID: 2724096.
5. Duggan KC, Walters MJ, Musee J, Harp JM, Kiefer JR, Oates JA, et al. Molecular basis for cyclooxygenase inhibition by the non-steroidal anti-inflammatory drug naproxen. J Biol Chem. 2010;285(45):34950-9. doi: 10.1074/jbc.M110.162982. PubMed PMID: 20810665; PubMed Central PMCID: PMC2966109.
6. Barnett J, Chow J, Ives D, Chiou M, Mackenzie R, Osen E, et al. Purification, characterization and selective inhibition of human prostaglandin G/H synthase 1 and 2 expressed in the baculovirus system. Biochim Biophys Acta. 1994;1209(1):130-9. PubMed PMID: 7947975.
7. Laneuville O, Breuer DK, Dewitt DL, Hla T, Funk CD, Smith WL. Differential inhibition of human prostaglandin endoperoxide H synthases-1 and -2 by nonsteroidal anti-inflammatory drugs. J Pharmacol Exp Ther. 1994;271(2):927-34. PubMed PMID: 7965814.
8. Huntjens DR, Spalding DJ, Danhof M, Della Pasqua OE. Correlation between in vitro and in vivo concentration-effect relationships of naproxen in rats and healthy volunteers. Br J Pharmacol. 2006;148(4):396-404. doi: 10.1038/sj.bjp.0706737. PubMed PMID: 16682968; PubMed Central PMCID: PMC1751780.
9. Kato M, Nishida S, Kitasato H, Sakata N, Kawai S. Cyclooxygenase-1 and cyclooxygenase-2 selectivity of non-steroidal anti-inflammatory drugs: investigation using human peripheral monocytes. J Pharm Pharmacol. 2001;53(12):1679-85. PubMed PMID: 11804398.
10. Patrignani P, Panara MR, Greco A, Fusco O, Natoli C, Iacobelli S, et al. Biochemical and pharmacological characterization of the cyclooxygenase activity of human blood prostaglandin endoperoxide synthases. J Pharmacol Exp Ther. 1994;271(3):1705-12. PubMed PMID: 7996488.
11. Riendeau D, Percival MD, Boyce S, Brideau C, Charleson S, Cromlish W, et al. Biochemical and pharmacological profile of a tetrasubstituted furanone as a highly selective COX-2 inhibitor. Br J Pharmacol. 1997;121(1):105-17. doi: 10.1038/sj.bjp.0701076. PubMed PMID: 9146894; PubMed Central PMCID: PMC1564640.
12. Warner TD, Giuliano F, Vojnovic I, Bukasa A, Mitchell JA, Vane JR. Nonsteroid drug selectivities for cyclo-oxygenase-1 rather than cyclo-oxygenase-2 are associated with human gastrointestinal toxicity: a full in vitro analysis. Proc Natl Acad Sci U S A. 1999;96(13):7563-8. PubMed PMID: 10377455; PubMed Central PMCID: PMC22126.
13. Jett MF, Ramesha CS, Brown CD, Chiu S, Emmett C, Voronin T, et al. Characterization of the analgesic and anti-inflammatory activities of ketorolac and its enantiomers in the rat. J Pharmacol Exp Ther. 1999;288(3):1288-97. PubMed PMID: 10027870.
14. Handley DA, Cervoni P, McCray JE, McCullough JR. Preclinical enantioselective pharmacology of (R)- and (S)- ketorolac. J Clin Pharmacol. 1998;38(2 Suppl):25S-35S. PubMed PMID: 9549656.
15. Mroszczak E, Combs D, Chaplin M, Tsina I, Tarnowski T, Rocha C, et al. Chiral kinetics and dynamics of ketorolac. J Clin Pharmacol. 1996;36(6):521-39. PubMed PMID: 8809637.
16. Mills JEJ, P.M. D. Three-dimensional hydrogen-bond geometry and probability information from a crystal survey. J Comput Aided Mol Design. 1996;10:607-19.
17. Pini LA, Bertolotti M, Trenti T, Vitale G. Disposition of naproxen after oral administration during and between migraine attacks. Headache. 1993;33(4):191-4. PubMed PMID: 8496057.

18. Cohen A, Basch C. Steady state pharmacokinetics of naproxen in young and elderly healthy volunteers. Semin Arthritis Rheum. 1988;17(3 Suppl 2):7-11. PubMed PMID: 3508329.
19. Furst DE, Sarkissian E, Blocka K, Cassell S, Dromgoole S, Harris ER, et al. Serum concentrations of salicylate and naproxen during concurrent therapy in patients with rheumatoid arthritis. Arthritis Rheum. 1987;30(10):1157-61. PubMed PMID: 3675660.
20. Buckley MM, Brogden RN. Ketorolac. A review of its pharmacodynamic and pharmacokinetic properties, and therapeutic potential. Drugs. 1990;39(1):86-109. PubMed PMID: 2178916.
21. Kendall MJ, Chellingsworth MC, Jubb R, Thawley AR, Undre NA, Kill DC. A pharmacokinetic study of the active metabolite of nabumetone in young healthy subjects and older arthritis patients. Eur J Clin Pharmacol. 1989;36(3):299-305. PubMed PMID: 2744071.
22. Pallapies D, Peskar BA, Brune K, Geisslinger G. Effects on platelet functions and pharmacokinetics of azapropazone and ketorolac tromethamine given as single parenteral doses. Br J Clin Pharmacol. 1994;37(4):335-9. PubMed PMID: 8018454; PubMed Central PMCID: PMC1364733.
23. Kline GS. A Single Dose Study to Evaluate the Dose Proportionality of the Tablet Formulation of Nabumetone Q and the Relative Bioavailability of Nabumetone Q Compared to RELAFEN in Healthy Subjects. Study No: BRL-014777/268 [PDF]. Glaxo Smith Kline 2001 [June 30, 2014]. Available from: Suppl. Fig S1-S8 Tables S1-S7 references final revised with tiffs.docx Study BRL-014777/268.
24. Pallapies D, Salinger A, Meyer zum Gottesberge A, Atkins DJ, Rohleder G, Nagyivanyi P, et al. Effects of lysine clonixinate and ketorolac tromethamine on prostanoid release from various rat organs incubated ex vivo. Life Sci. 1995;57(2):83-9. PubMed PMID: 7603299.
25. Hinz B, Cheremina O, Besz D, Zlotnick S, Brune K. Impact of naproxen sodium at over-the-counter doses on cyclooxygenase isoforms in human volunteers. Int J Clin Pharmacol Ther. 2008;46(4):180-6. PubMed PMID: 18397691.
26. Kean WF, Lock CJ, Howard-Lock HE. Chirality in antirheumatic drugs. Lancet. 1991;338(8782-8783):1565-8. PubMed PMID: 1683980.
27. Gao Y, Dickerson JB, Guo F, Zheng J, Zheng Y. Rational design and characterization of a Rac GTPase-specific small molecule inhibitor. Proc Natl Acad Sci U S A. 2004;101(20):7618-23. doi: 10.1073/pnas.0307512101. PubMed PMID: 15128949; PubMed Central PMCID: PMC419655.
28. Surviladze Z, Young SM, Sklar LA. High-throughput flow cytometry bead-based multiplex assay for identification of Rho GTPase inhibitors. Methods Mol Biol. 2012;827:253-70. doi: 10.1007/978-1-61779-442-1_17. PubMed PMID: 22144280.
29. Corbeil CR, Williams CI, Labute P. Variability in docking success rates due to dataset preparation. J Comput Aided Mol Des. 2012;26(6):775-86. doi: 10.1007/s10822-012-9570-1. PubMed PMID: 22566074; PubMed Central PMCID: PMC3397132.
30. Labute P. The generalized Born/volume integral implicit solvent model: estimation of the free energy of hydration using London dispersion instead of atomic surface area. J Comput Chem. 2008;29(10):1693-8. doi: 10.1002/jcc.20933. PubMed PMID: 18307169.
